# SARS-CoV-2 nucleocapsid protein, rather than spike protein, triggers a cytokine storm originating from lung epithelial cells in patients with COVID-19

**DOI:** 10.1007/s15010-023-02142-4

**Published:** 2023-12-22

**Authors:** Ying‑Chuan Wang, Chih-Hsuan Tsai, Yung-Chih Wang, Li-Chen Yen, Yao-Wen Chang, Jun-Ren Sun, Te-Yu Lin, Chun-Hsiang Chiu, Yu-Chan Chao, Feng-Yee Chang

**Affiliations:** 1 Department of Family Medicine, Tri-Service General Hospital, National Defense Medical Center, No. 161, Sec. 6, Minquan E. Rd., Neihu Dist., Taipei City, 11499 Taiwan, ROC; 2 Department of Microbiology and Immunology, College of Medicine, National Cheng Kung University, Tainan, 701 Taiwan, ROC; 3 Division of Infectious Diseases and Tropical Medicine, Department of Internal Medicine, Tri-Service General Hospital, National Defense Medical Center, No. 161, Sec. 6, Minquan E. Rd., Neihu Dist., Taipei City, 11499 Taiwan, ROC; 4 Department of Microbiology and Immunology, National Defense Medical Center, No. 161, Sec. 6, Minquan E. Rd., Neihu Dist., Taipei City, 11499 Taiwan, ROC; 5 Taoyuan Armed Forces General Hospital, Taoyuan, 32551 Taiwan, ROC; 6 Institute of Preventive Medicine, National Defense Medical Center, No. 161, Sec. 6, Minquan E. Rd., Neihu Dist., Taipei City, 11499 Taiwan, ROC; 7 Department of Entomology, College of Agriculture and Nature Resources, National Chung Hsing University, Taichung, 40227 Taiwan, ROC

**Keywords:** COVID-19, Cytokine storm, IP-10, Nucleocapsid protein, SARS-CoV-2

## Abstract

**Purpose:**

The aim of this study was to elucidate the factors associated with severe acute respiratory syndrome coronavirus 2 (SARS-CoV-2) that may initiate cytokine cascades and correlate the clinical characteristics of patients with coronavirus disease 2019 (COVID-19) with their serum cytokine profiles.

**Methods:**

Recombinant baculoviruses displaying SARS-CoV-2 spike or nucleocapsid protein were constructed and transfected into A549 cells and THP-1-derived macrophages, to determine which protein initiate cytokine release. SARS-CoV-2-specific antibody titers and cytokine profiles of patients with COVID-19 were determined, and the results were associated with their clinical characteristics, such as development of pneumonia or length of hospital stay.

**Results:**

The SARS-CoV-2 nucleocapsid protein, rather than the spike protein, triggers lung epithelial A549 cells to express IP-10, RANTES, IL-16, MIP-1α, basic FGF, eotaxin, IL-15, PDGF-BB, TRAIL, VEGF-A, and IL-5. Additionally, serum CTACK, basic FGF, GRO-α, IL-1α, IL-1RA, IL-2Rα, IL-9, IL-15, IL-16, IL-18, IP-10, M-CSF, MIF, MIG, RANTES, SCGF-β, SDF-1α, TNF-α, TNF-β, VEGF, PDGF-BB, TRAIL, β-NGF, eotaxin, GM-CSF, IFN-α2, INF-γ, and MCP-1 levels were considerably increased in patients with COVID-19. Among them, patients with pneumonia had higher serum IP-10 and M-CSF levels than patients without. Patients requiring less than 3 weeks to show negative COVID-19 tests after contracting COVID-19 had higher serum IP-10 levels than the remaining patients.

**Conclusion:**

Our study revealed that nucleocapsid protein, lung epithelial cells, and IP-10 may be potential targets for the development of new strategies to prevent, or control, severe COVID-19.

**Supplementary Information:**

The online version contains supplementary material available at 10.1007/s15010-023-02142-4.

## Introduction

Coronavirus disease 2019 (COVID-19) is an ongoing global pandemic caused by severe acute respiratory syndrome coronavirus 2 (SARS-CoV-2) of which pneumonia is the most common complication followed by acute respiratory distress syndrome (ARDS) [1]. The severity of COVID-19 is directly linked to the cytokine storm triggered by SARS-CoV-2 [2, 3]. However, data on the mechanism of these virulent factors of SARS-CoV-2, in addition to COVID-19 pathoetiology, remain lacking.

A cytokine storm, an activation cascade resulting from the production of auto-amplifying cytokines and chemokines, involves the accumulation of hyperactive immune cells due to an unregulated immune response [4]. The cytokine storm process may involve numerous immune cells of different types, epithelial cells, fibrocytes, and cytokines. Indeed, several cytokines have been found to be upregulated in patients with COVID-19. Identifying these participating cytokines and their associated receptors which are related to the clinical outcomes is essential for targeted therapeutics to mitigate disease severity in patients with COVID-19. Pneumonia is the most common complication of COVID-19 [1], affecting the progression of the disease. The severity of COVID-19 and treatment guidelines are even classified based on the presence and extent of pneumonia as follows [5]: patients without pneumonia are classified as mild cases; patients with pneumonia but without hypoxemia (oxygen saturation > 94%) as moderate disease cases; patients with pneumonia who are in respiratory distress or hypoxemia (oxygen saturation ≤ 94%) are classified as severe cases; and patients with pneumonia who are in respiratory failure, shock, or failure of other organs requiring intensive care unit are classified as critical cases [5]. Given the significant impact of pneumonia on the clinical outcomes of patients with COVID-19, in this study, we explored the correlation between cytokine expression and clinical characteristics, particularly the development of pneumonia or duration of hospitalization, in patients with COVID-19.

In contrast to the pleomorphic and diverse serum cytokines and participating immune cells in patients with severe COVID-19, the factors and cytokines involved in the initial stages of the cytokine cascades may be limited in number and less complicated. Therefore, targeting the virulence factors of SARS-CoV-2, the participating immune cells, and secreted cytokines during the initial stage of cytokine cascades, may be an efficient strategy to prevent the progression of severe COVID-19. Therefore, we performed an in vitro study, in A549 and THP-1 cells, to identify these important factors.

## Materials and methods

### Cell lines and media

*Spodoptera frugiperda* IPLB-Sf21 (Sf21) cells, used for amplifying baculoviruses, were cultured at 26 °C in TC100 insect medium (Thermo Fisher Scientific, Waltham, MA, USA) supplemented with 10% fetal bovine serum (FBS). Human lung adenocarcinoma (A549) and human leukemia monocytic (THP-1) cell lines were cultured in a humidified incubator at 37 °C with 5% CO_2_. A549 cells were maintained in Ham's F-12 K (Kaighn's) medium (Invitrogen, Carlsbad, CA, USA) and THP-1 cells were cultured in RPMI 1640 (Sigma-Aldrich, St. Louis, MO, USA); both media were supplemented with 10% FBS and 100 units/mL of penicillin/streptomycin (Thermo Fisher Scientific). To induce differentiation in macrophages, 40 ng/mL phorbol 12-myristate 13-acetate (PMA; Sigma-Aldrich) was added to THP-1 cell cultures for 36 h.

### Recombinant baculovirus construction

The baculovirus expression vector system is an efficient tool for producing heterologous recombinant proteins [6]. The resulting expressed recombinant proteins are often soluble, folded correctly, and biologically active [7]. We constructed S-Bac and N-Bac recombinant baculoviruses displaying the SARS-CoV-2 spike (S) and nucleocapsid (N) proteins, respectively, based on a previous study [8]. EG-Bac, the recombinant baculovirus expressing enhanced green fluorescent protein, was constructed as a control vector as previously described [9]. In brief, the pTriEx-4 plasmid (MilliporeSigma, Burlington, MA, USA), harboring the mCherry reporter gene, driven by *SV40* and *pag* promoters [10], was used as a cloning vector. We sequentially cloned the poly-histidine tag (honeybee melittin signal peptide nucleotide sequence), baculovirus GP64 transmembrane domain (TMD), and cytoplasmic tail domain downstream of TriEx promoter in the vector. The S and N nucleotide sequences were synthesized based on the Wuhan-Hu-1 isolate (GenBank accession No: MN908947.3; Mission BioTec, Taiwan). The ectodomain of S and full-length N were inserted between the poly-histidine tag and GP64 TMD. Cloning was performed using the In-Fusion^®^ HD Cloning Kit (Takara Bio, San Jose, CA, USA). The resulting plasmids were co-transfected with baculoviral DNA FlashBAC™ (Mirus Bio, Madison, WI, USA) into Sf21 cells. The recombinant viruses were harvested from the culture supernatant after 5 d of incubation at 26 °C. Individual viruses were selected through serial dilution; the recombinant protein expression and surface protein expression were verified through Western blotting.

### Inoculation of recombinant baculoviruses into cell models

After the viral titers were determined via quantitative polymerase chain reaction [11], the three different recombinant baculoviruses were added, respectively, to A549 cells and THP-1-derived macrophages, at a multiplicity of infection of 200. The cells were then seeded in 24-well plates (5 × 10^4^/well) and cultured at 37 °C and 5% CO_2_. Culture media were collected at 3-, 6-, 12-, 24-, and 48 h post-inoculation and centrifuged at 700 × *g* for 30 min at 25 °C. Thereafter, the supernatant was subjected to cytokine analysis using the Bio-Plex Pro Human Cytokine Screening Panel 48-Plex (#12007283; Bio-Rad, Hercules, CA, USA), according to the manufacturer's instructions. Independent experiments for each condition—cell line, virus treatment, and treatment time—were performed in triplicate.

### Polarization of THP-1-derived macrophages before and after inoculation with S-Bac or N-Bac

After the incubation with EG-Bac, S-Bac, and N-Bac, respectively, total RNA of the PMA-differentiated THP-1 macrophages was extracted at 0 and 48 h post-transduction using the Quick-RNATM MiniPrep kit (Zymo Research, R1054). The extracted RNA was reverse transcribed using complementary DNA (cDNA) using random hexamer and M-MLV transcriptase (Promega, M1705). Amplification reaction assays contained GoTaq^®^ SYBR Green qPCR Master Mix (Promega, A6002) and primers listed in Table 1. GAPDH was used as the reference gene for normalization and mRNA expression was quantified using the 2^−ΔΔCT^ method.

**Table 1:** Primers used for characterization of macrophage polarization

Primers	DNA sequence (5′ → 3′)
hCD86 Forward	ACATTCTCTTTGTGATGGCCTTC
hCD86 Reverse	CCAAGTTTTCCTGGTCCTGC
hCD204 Forward	AGTGCTGCTTTCTTTAGGACG
hCD204 Reverse	GGATTCGGAGGAAGCAAAGC
hIL-6 Forward	TGTGAAAGCAGCAAAGAGGCACTG
hIL-6 Reverse	ACAGCTCTGGCTTGTTCCTCACTA
hTGF-β Forward	CTCGCCAGAGTGGTTATCTT
hTGF-β Reverse	AGTGTGTTATCCCTGCTGTCA

### Patients and blood samples collection

At the Tri-Service General Hospital, Northern Taiwan, adult patients (≥ 18 years old) with COVID-19, hospitalized between April 2020 and August 2020, who were willing to participate in our study, were enrolled. Patients who were unclear, had trouble expressing their willingness to participate, or those who were not willing to participate were excluded. Ten healthy adult volunteers (≥ 18 years old) were enrolled as control group. The diagnosis of these patients was confirmed based on clinical indications and reverse transcriptase polymerase chain reaction. Pneumonia was confirmed via chest radiography or computed tomography. Patients were discharged according to the criteria decided by the Taiwan’s Centers for Disease Control, which require symptoms to have subsided and three negative test results for COVID-19 from oropharyngeal swab specimens and sputum to have been obtained. This study was approved by the Institutional Review Board of Tri-Service General Hospital (C202005067), and informed written consent was obtained from the included participants.

To investigate the COVID-19 pathogenesis, serum samples of patients were collected every week and subjected to cytokine and immunoglobulin analyses. Patients were classified based on the presence or absence of pneumonia and their duration of hospitalization (hospitalization ≥ 21 d vs. hospitalization < 21 d). The serum antibody levels and cytokine expression between the different groups of patients were compared.

### Cytokine and immunoglobulin analyses

Cytokine profiles of serum samples were analyzed using Bio-Plex Pro Human Cytokine Screening Panel 48-Plex as described above. SARS-CoV-2 IgG and IgM titers were determined using Anti-SARS-CoV-2 S receptor-binding domain (RBD) protein (wild-type) Human IgG ELISA Kit (KE30003; Proteintech, Rosemont, IL, USA), Anti-SARS-CoV-2 S-RBD protein (wild-type) Human IgM ELISA Kit (KE30004; Proteintech), Anti-SARS-CoV-2 N protein (wild-type) Human IgG (Proteintech, Catalog No. KE30001), and Anti-SARS-CoV-2 N protein (wild-type) Human IgM (Proteintech, Catalog No. KE30002), respectively, according to the manufacturer's instructions. Antibody levels were calculated using the established standard curves. The levels of each cytokine in healthy subjects, indicated as dotted lines in the figures of our manuscript, were according to the previous literatures [12, 13].

### Statistical analyses

For the in vitro study, the cytokine expression levels produced by stimulating A549 cells or THP-1 cells with S-bac or N-bac, respectively, were compared with those produced by stimulating A549 or THP-1 cells with the control, EG-bac. For the two-group comparisons, independent *t*-test was performed. In assessing THP-1 polarization, we compared the expression levels of markers or cytokines for THP-1 polarization before and 48 h after stimulation using a paired *t*-test. For the clinical assessment, the serum cytokine levels collected weekly from patients with COVID-19 starting from the onset of symptoms were measured and compared with those in healthy controls using an independent *t*-test. The comparison of serum antibody or cytokine levels of COVID-19 patients subgrouped based on pneumonia diagnosis or duration of hospitalization (≥ 21 d vs. < 21 d) was conducted using an independent *t-*test. Data are expressed as means ± standard deviations. All statistical analyses were performed using GraphPad Prism 9 (GraphPad Software Inc.). Statistical significance was set at *p* < 0.05.

## Results

### Cytokines level secreted by A549 cells

**Fig. 1:**
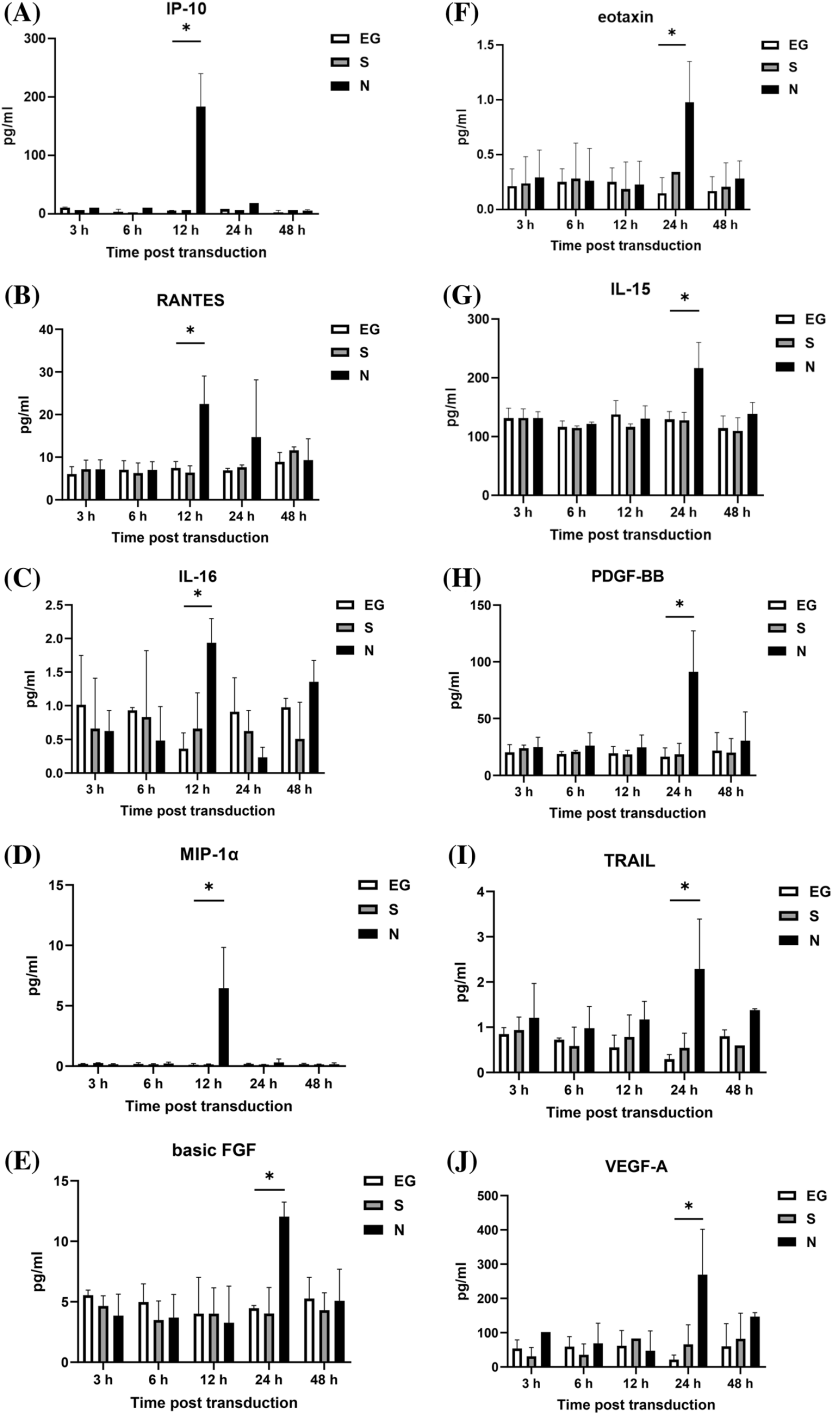

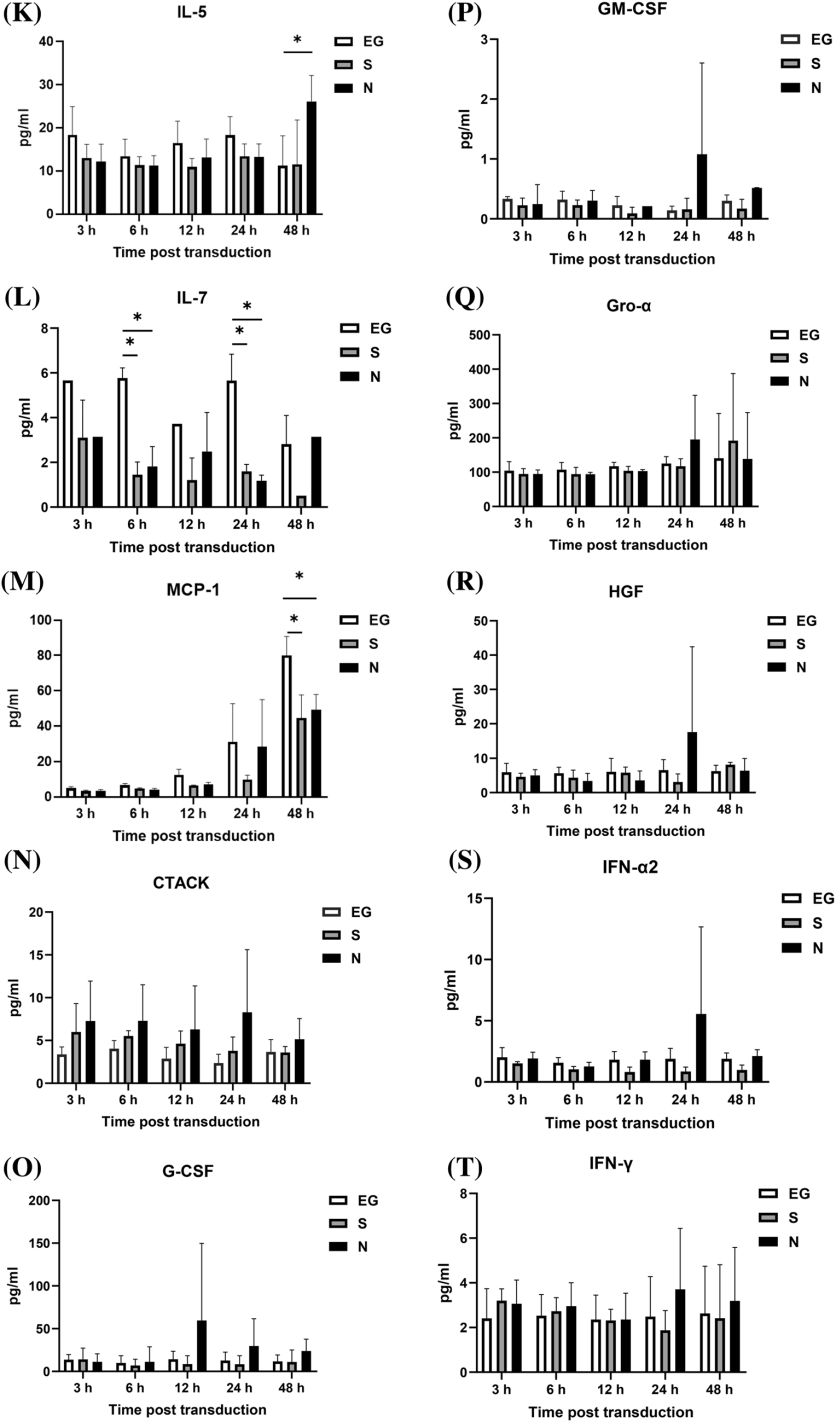

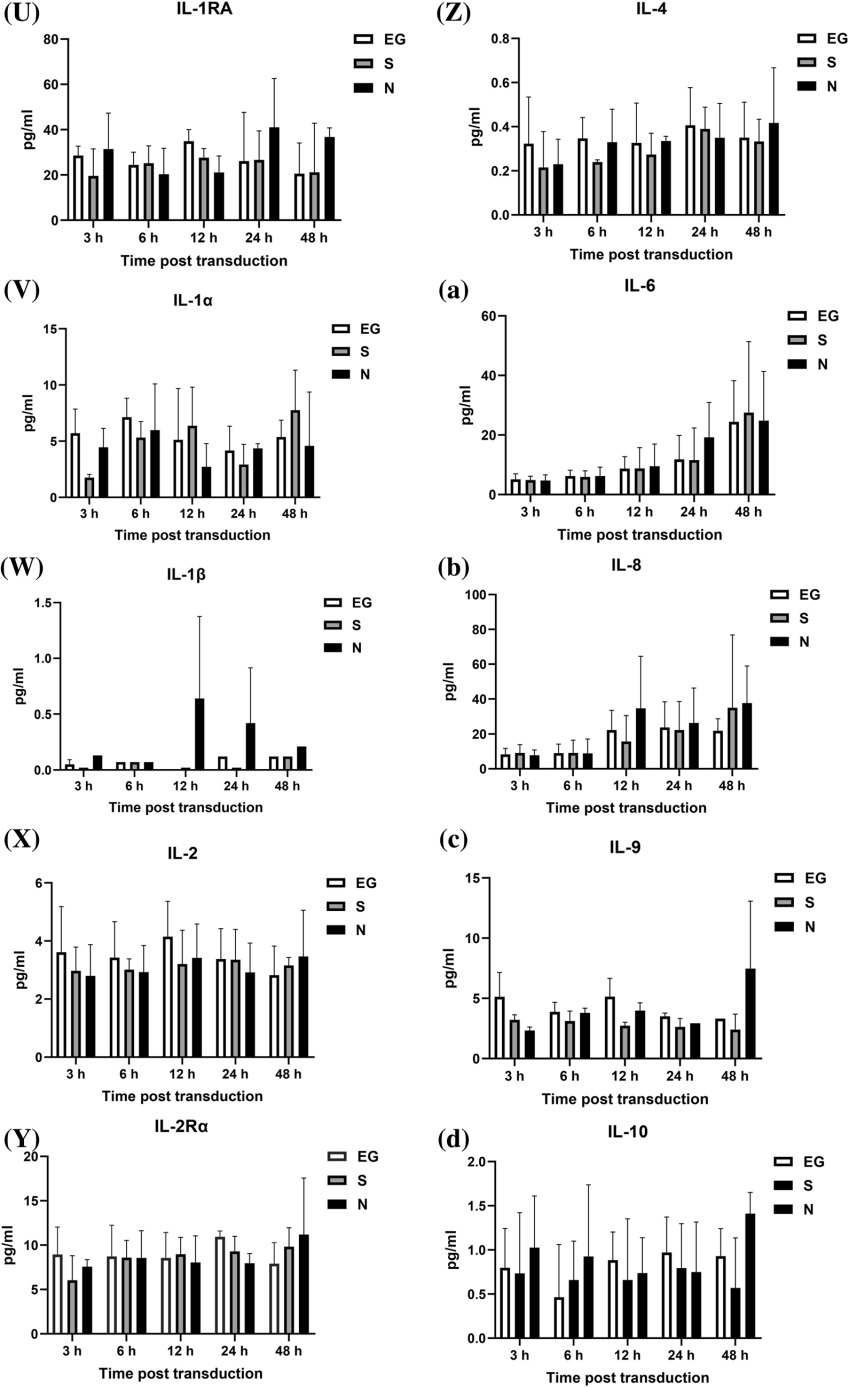

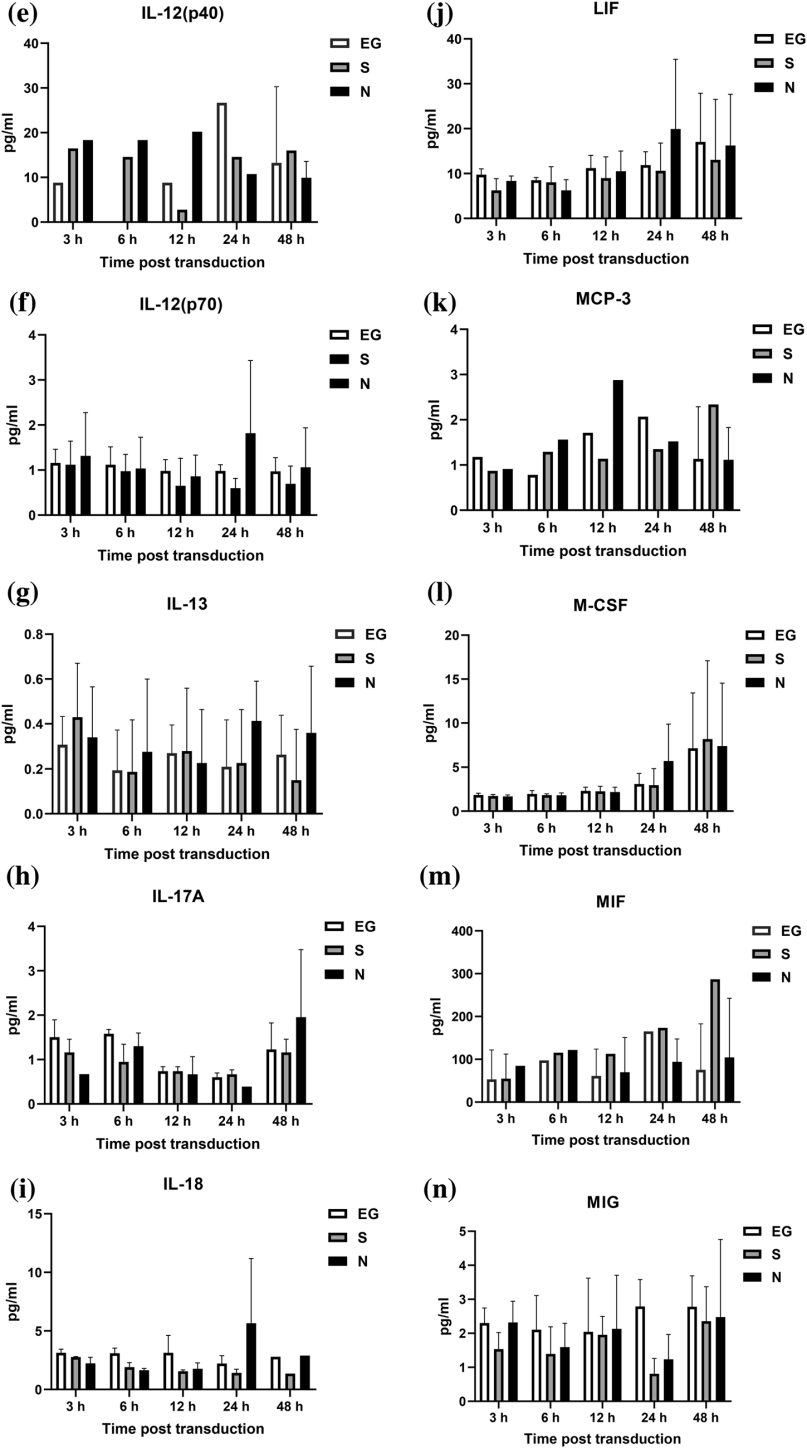

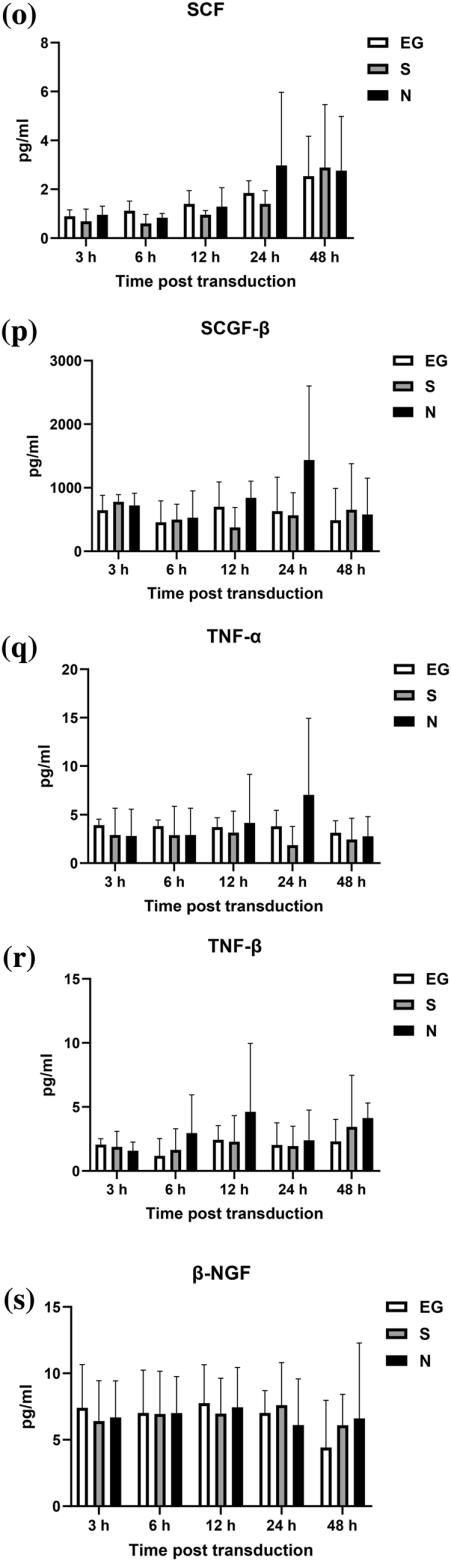
Expression levels of cytokines secreted by A549 cells after inoculation with EG-Bac, S-Bac, or N-Bac, respectively, over 3, 6, 12, 24, and 48 h. The cytokine expression levels produced by stimulating A549 cells with S-bac or N-bac were compared with those produced by stimulating with the control, EG-bac. *p < 0.05

The cytokines secreted by A549 cells, after inoculated with EG-Bac, S-Bac, or N-Bac over 3-, 6-, 12-, 24-, and 48 h, are shown in Fig. 1. Compared with the cells inoculated with EG-Bac, cells inoculated with N-Bac expressed significantly higher levels of IP-10 (N-Bac vs. EG-Bac; 183.64 ± 56.64 vs. 4.98 ± 1.00 pg/mL; *p* = 0.005), RANTES (N-Bac vs. EG-Bac; 22.48 ± 6.57 vs. 7.49 ± 1.49 pg/mL; *p* = 0.018), IL-16 (N-Bac vs. EG-Bac; 1.94 ± 0.36 vs. 0.36 ± 0.24 pg/mL; *p* = 0.003), MIP-1α (N-Bac vs. EG-Bac; 6.46 ± 3.37 vs. 0.12 ± 0.10 pg/mL; *p* = 0.031), basic FGF (N-Bac vs. EG-Bac; 12.05 ± 1.20 vs. 4.47 ± 0.23 pg/mL; *p* < 0.001), eotaxin (N-Bac vs. EG-Bac; 0.98 ± 0.37 vs. 0.15 ± 0.14 pg/mL; *p* = 0.022), IL-15 (N-Bac vs. EG-Bac; 217.32 ± 43.26 vs. 129.71 ± 13.40 pg/mL; *p* = 0.029), PDGF-BB (N-Bac vs. EG-Bac; 91.24 ± 36.06 vs. 16.54 ± 7.79 pg/mL; *p* = 0.025), TRAIL (N-Bac vs. EG-Bac; 2.29 ± 1.11 vs. 0.29 ± 0.10 pg/mL; *p* = 0.035), VEGF-A (N-Bac vs. EG-Bac; 269.28 ± 132.63 vs. 21.29 ± 13.50 pg/mL; *p* = 0.032), and IL-5 (N-Bac vs. EG-Bac; 26.09 ± 6.07 vs. 11.18 ± 6.99 pg/mL; *p* = 0.049) (Fig. 1A–K). A549 cells inoculated with N-Bac or S-Bac expressed significantly lower IL-7 levels than those with EG-Bac 6 h (N-Bac vs. EG-Bac; 1.82 ± 0.89 vs. 5.77 ± 0.45 pg/mL; *p* = 0.002) (S-Bac vs. EG-Bac; 1.45 ± 0.56 vs. 5.77 ± 0.45 pg/mL; *p* < 0.001) and 24 h (N-Bac vs. EG-Bac; 1.18 ± 0.26 vs. 5.65 ± 1.19 pg/mL; *p* = 0.019) (S-Bac vs. EG-Bac; 1.59 ± 0.32 vs. 5.65 ± 1.19 pg/mL; *p* = 0.005) after inoculation (Fig. 1L). A549 cells inoculated with N-Bac or S-Bac for 48 h expressed significantly lower MCP-1 levels (N-Bac vs. EG-Bac; 49.20 ± 8.71 vs. 80.11 ± 10.70 pg/mL; *p* = 0.018) (S-Bac vs. EG-Bac; 44.64 ± 12.99 vs. 80.11 ± 10.70 pg/L *p* = 0.022) than those with EG-Bac (Fig. 1M). The levels of other cytokines did not substantially change in A549 cells after inoculation with N-Bac, S-Bac, or EG-Bac (*p* > 0.05).

### Cytokines level secreted by THP-1 cells

**Fig. 2:**
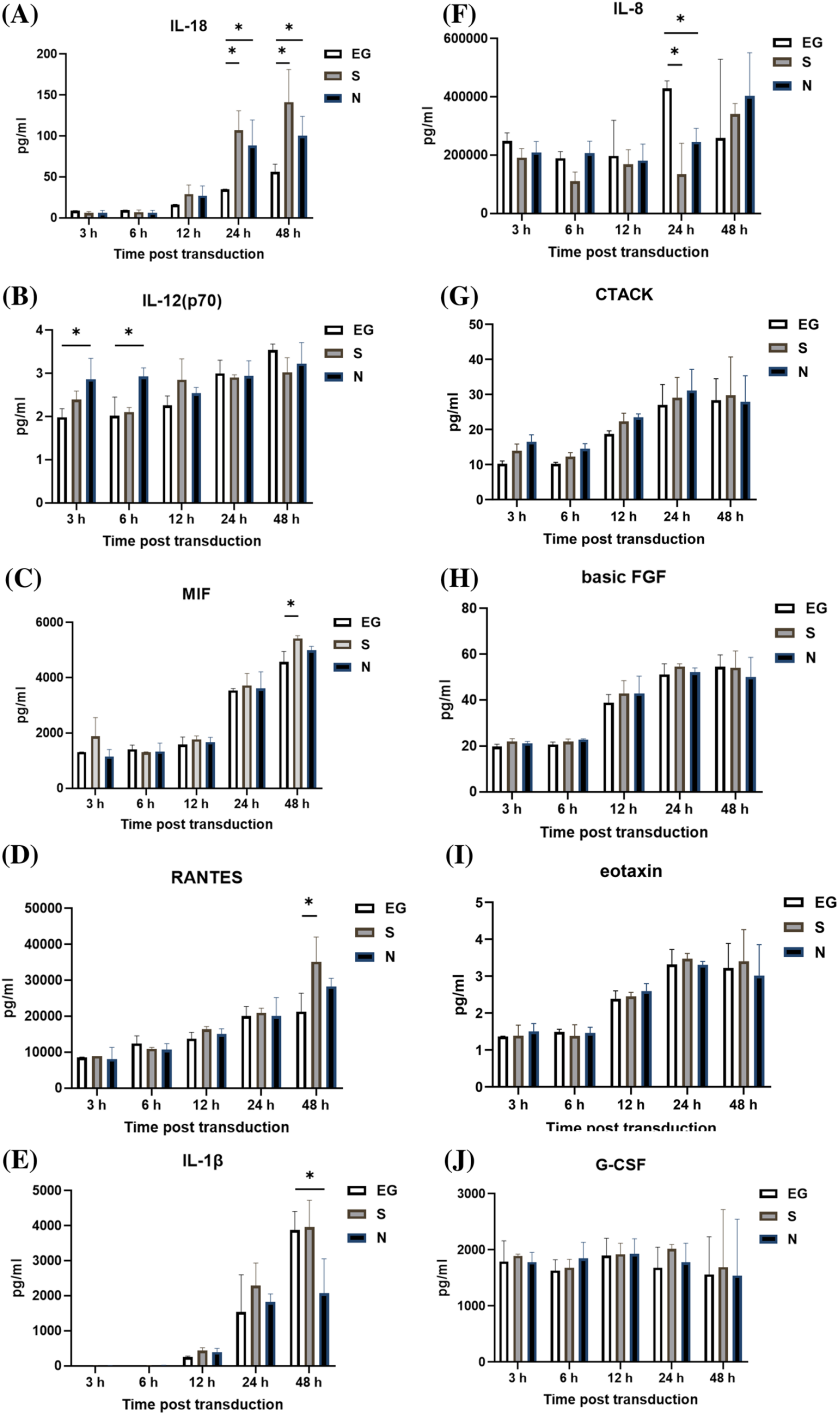

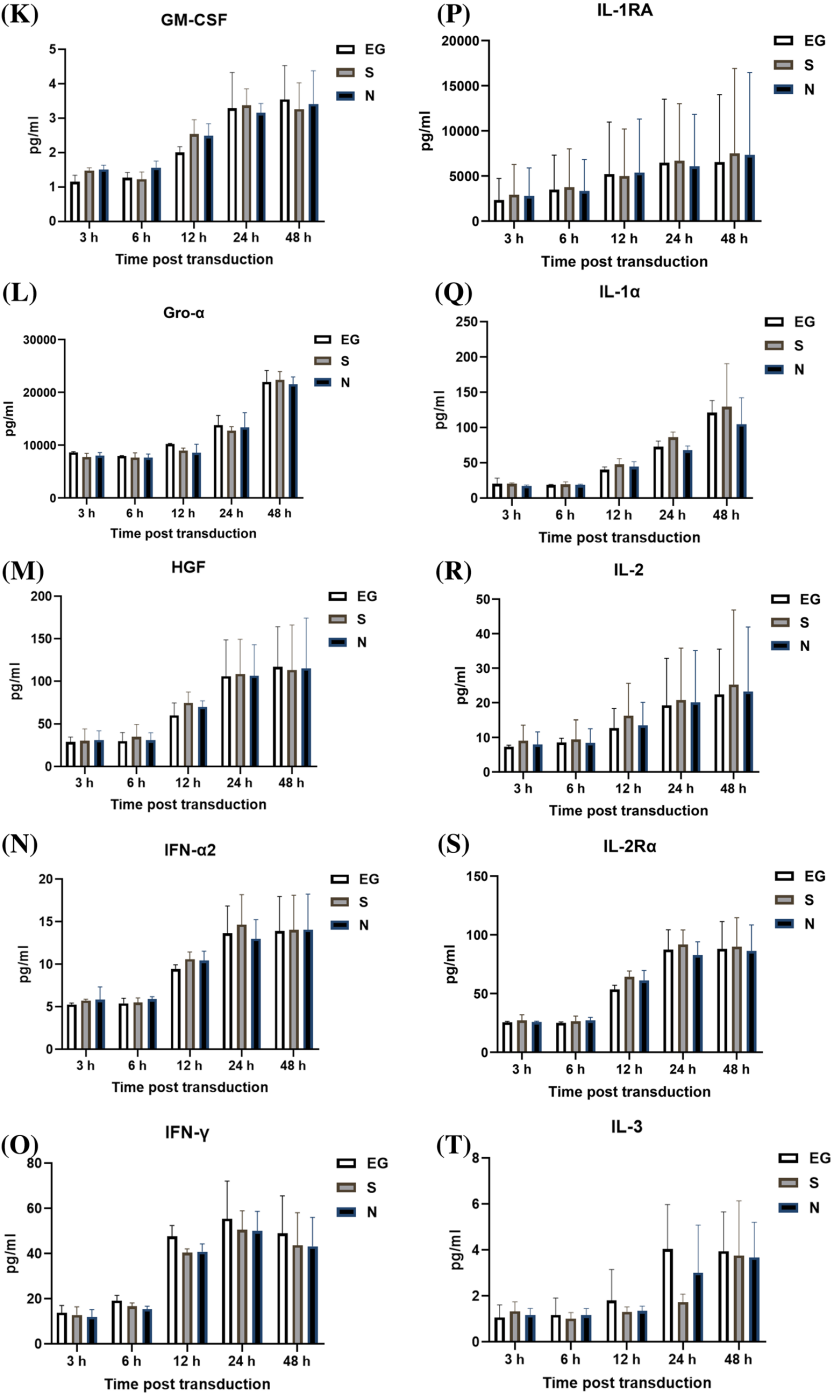

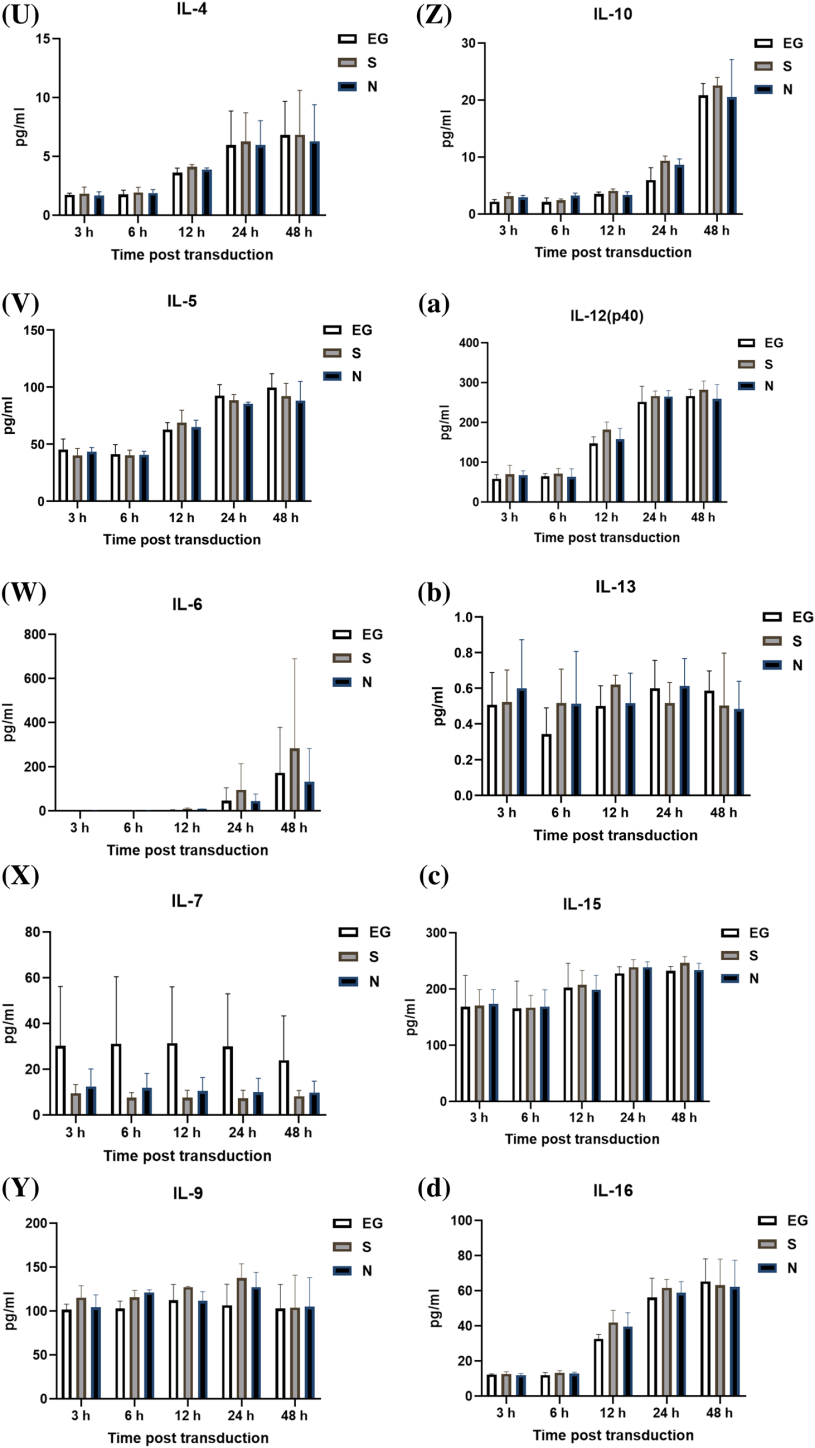

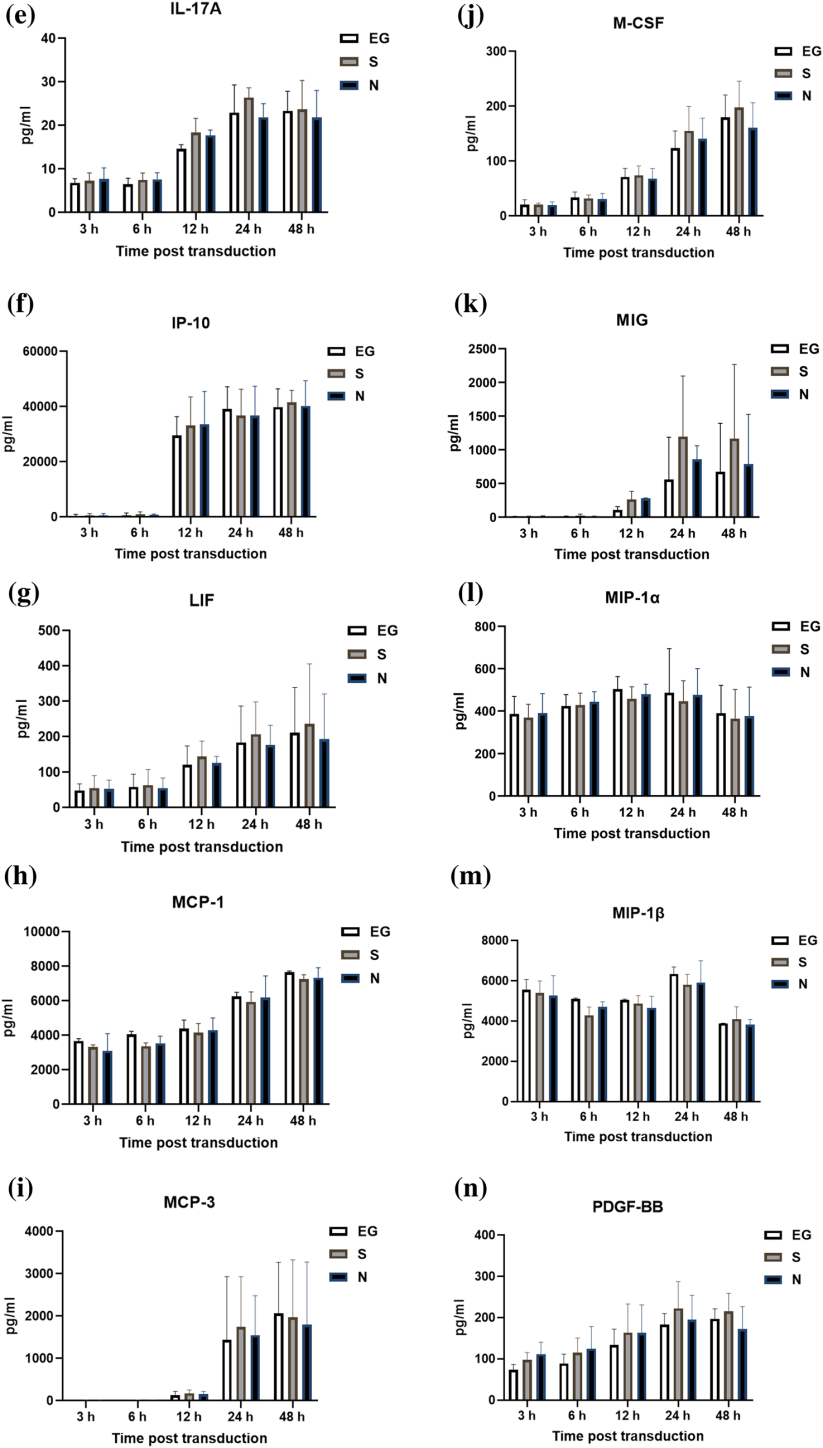

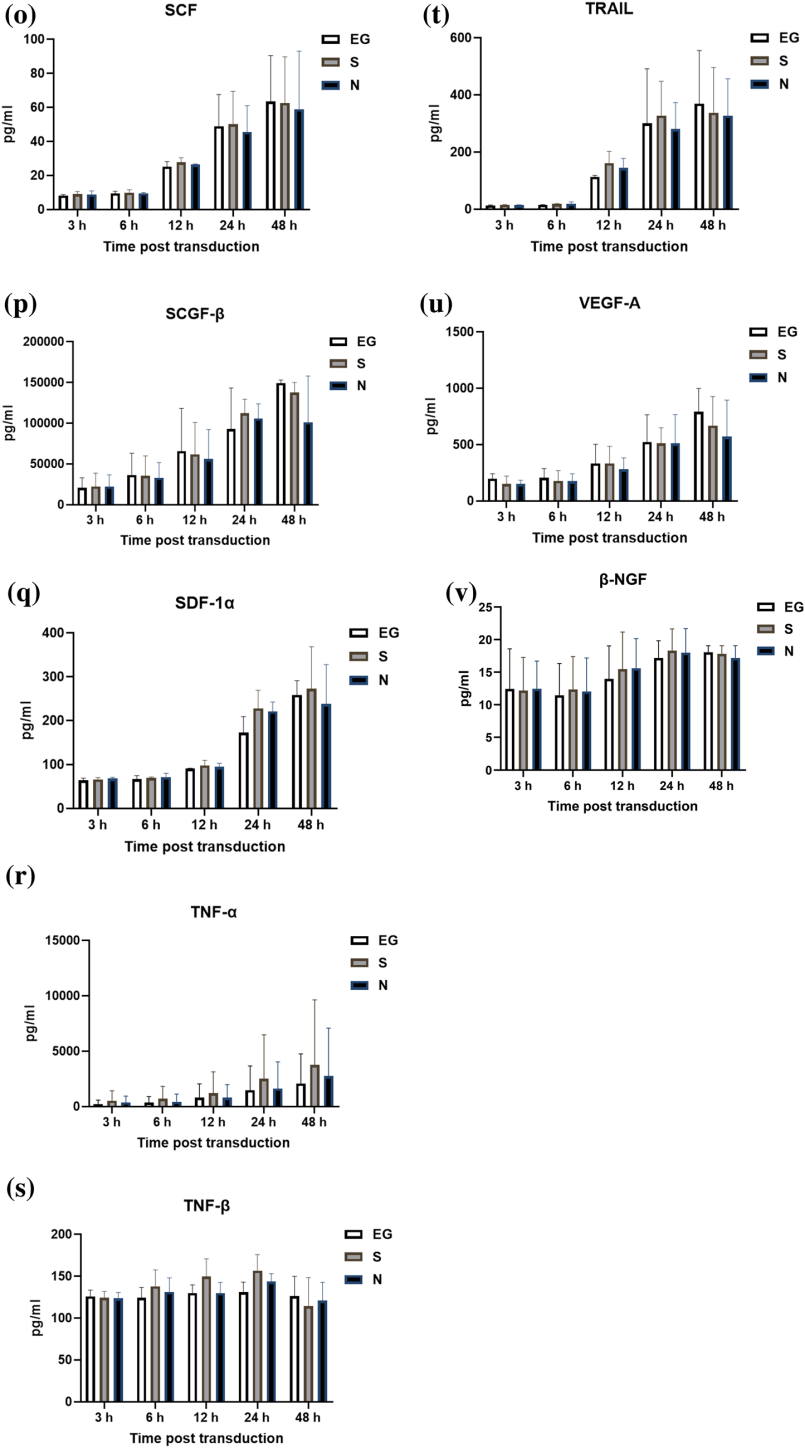
Expression levels of cytokines secreted by THP-1 cells after inoculation with EG-Bac, S-Bac, or N-Bac, respectively, over 3, 6, 12, 24, and 48 h. The cytokine expression levels produced by stimulating THP-1 cells with S-bac or N-bac were compared with those produced by stimulating with the control, EG-bac. *p < 0.05

The cytokines secreted by THP-1 cells, after inoculation with EG-Bac, S-Bac, or N-Bac over 3-, 6-, 12-, 24-, and 48 h, are shown in Fig. 2. THP-1 cells inoculated with N-Bac expressed significantly higher levels of IL-18 (N-Bac vs. EG-Bac; 88.25 ± 31.11 vs. 34.59 ± 0.52 pg/mL; *p* = 0.04) and IL-12 (P70) (N-Bac vs. EG-Bac; 2.87 ± 0.48 vs. 1.99 ± 0.20 pg/mL; *p* = 0.042) than those with EG-Bac (Fig. 2A, B). THP-1 cells inoculated with S-Bac expressed significantly higher levels of IL-18 (S-Bac vs. EG-Bac; 106.97 ± 23.81 vs. 34.59 ± 0.52 pg/mL; *p* = 0.034), MIF (S-Bac vs. EG-Bac; 5475.77 ± 125.22 vs. 4515.47 ± 282.83 pg/mL; *p* = 0.006), and RANTES (S-Bac vs. EG-Bac; 35,155.70 ± 6854.08 vs. 21,332.67 ± 5057.28 pg/mL; *p* = 0.048) compared with those inoculated with EG-Bac (Fig. 2A, C, D). Compared with THP-1 cells with EG-Bac, after 48 h, cells with N-Bac expressed significantly lower levels of IL-1β (N-Bac vs. EG-Bac; 2067.72 ± 983.27 vs. 3875.27 ± 532.98 pg/mL; *p* = 0.049) and IL-8 (N-Bac vs. EG-Bac; 245,177.67 ± 46,381.86 vs. 428,613 ± 26,274.93 pg/mL; *p* = 0.004) (Fig. 2E, F). Compared with THP-1 cells inoculated with EG-Bac, after 24 h, cells inoculated with S-Bac expressed significantly lower level of IL-8 (S-Bac vs. EG-Bac; 134,220.33 ± 106,449.00 vs. 428,613 ± 26,274.93 pg/mL; *p* = 0.01) (Fig. 2F). No change was observed in the expression levels of other cytokines in THP-1 cells inoculated with N-Bac, S-Bac, or EG-Bac.

### Markers and cytokines level for the polarization of THP-1 cells

**Fig. 3:**
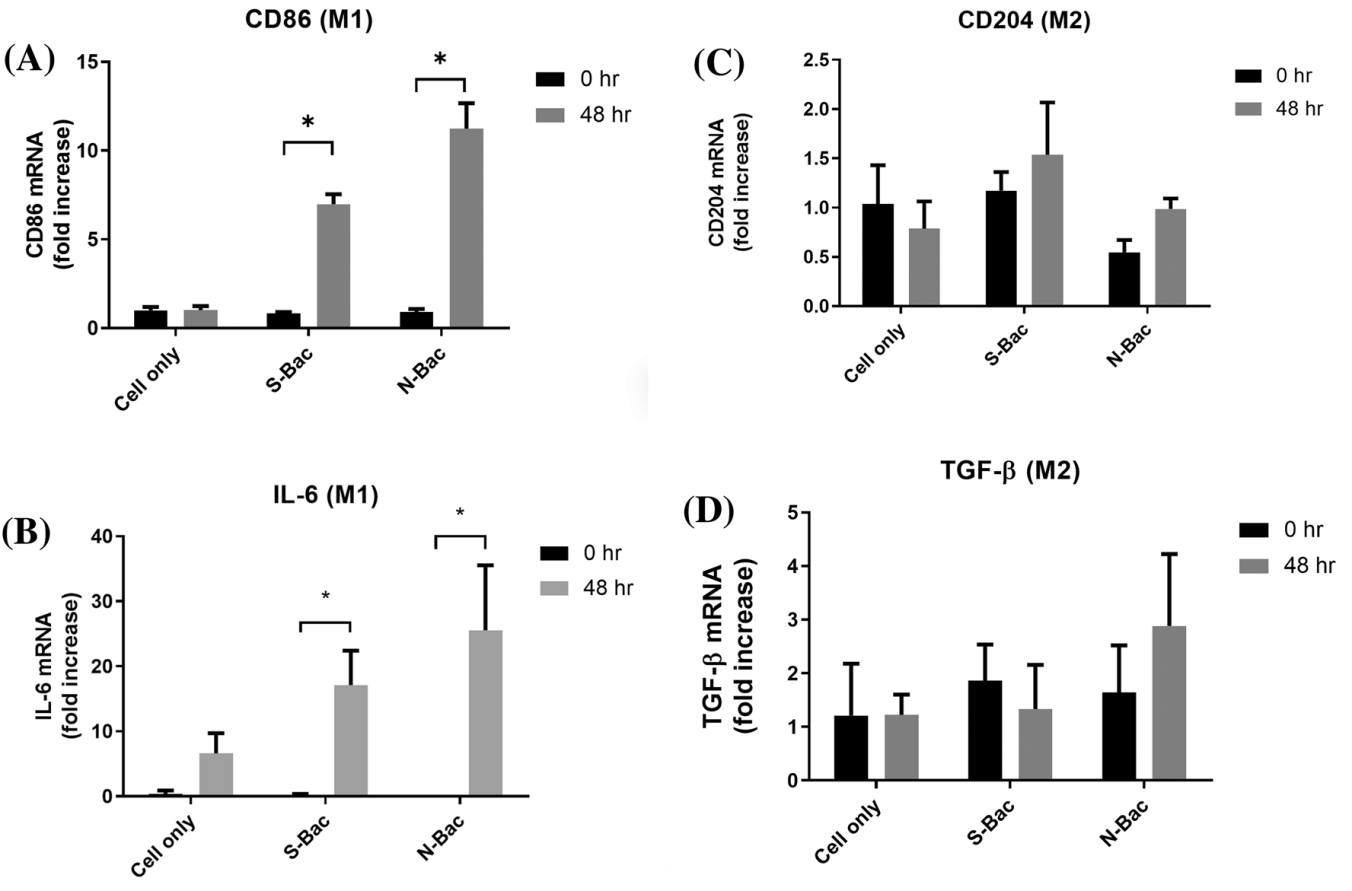
Expression levels of markers or cytokines for M1 polarization, including CD-86 (**A**) and IL-6 (**B**), and M2 polarization, including CD-204 (**C**) and TGF-β (**D**), were measured in THP-1 cells before and 48 h after inoculation with S-Bac or N-Bac. The expression levels of markers or cytokines for THP-1 polarization measured before and 48 h post-stimulation were compared using a pair *t*-test. *p < 0.05

The expression levels of markers or cytokines for M1 and M2 polarization, CD-86, IL-6, CD-204, and TGF-β expressed by THP-1 cells before or 48 h after inoculation with S-Bac or N-Bac, respectively, are shown in Fig. 3. After stimulation with S-Bac (48 h vs. 0 h; 6.98 ± 0.57 vs. 0.84 ± 0.06 fold; *p* = 0.0029), which was similar to that with N-Bac (48 h vs. 0 h; 11.24 ± 1.42 vs. 0.90 ± 0.19 fold; *p* = 0.0078), the expression level of CD-86 in THP-1 cells significantly increased 48 h post-stimulation compared with before stimulation (Fig. 3A). After stimulation with S-Bac (48 h vs. 0 h; 17.10 ± 5.30 vs. 0.20 ± 0.19 fold; *p* = 0.0334), which was similar to that with N-Bac (48 h vs. 0 h; 25.54 ± 10.01 vs. 0 ± 0 fold; *p* = 0.0476), the expression level of IL-6 in THP-1 cells significantly increased 48 h post-stimulation compared with before stimulation (Fig. 3B). Stimulating THP-1 cells with S-Bac or N-Bac did not significantly change the expression levels of CD-204 and TGF-β in THP-1 cells 48 h post-stimulation compared with before stimulation (Fig. 3C, D).

### Enrolled patients with COVID-19

Between April 2020 and August 2020, 27 patients with COVID-19 hospitalized at the Tri-Service General Hospital, and 10 healthy volunteers, were enrolled in our study. All patients were infected with wild-type SARS-CoV-2 and their serum samples were tested for anti-SARS-CoV-2 S-RBD/N protein IgM, IgG, and cytokine levels. The median age of the patients was 40 years (range 18–80 years), and the median Charlson comorbidity index was 0.93. Of the 27 enrolled patients, 17 (62.96%) had pneumonia; all patients recovered and were discharged, with a median hospital quarantine duration of 27.81 d.

### IgM, IgG, and cytokine levels in serum of patients with COVID-19

**Fig. 4:**
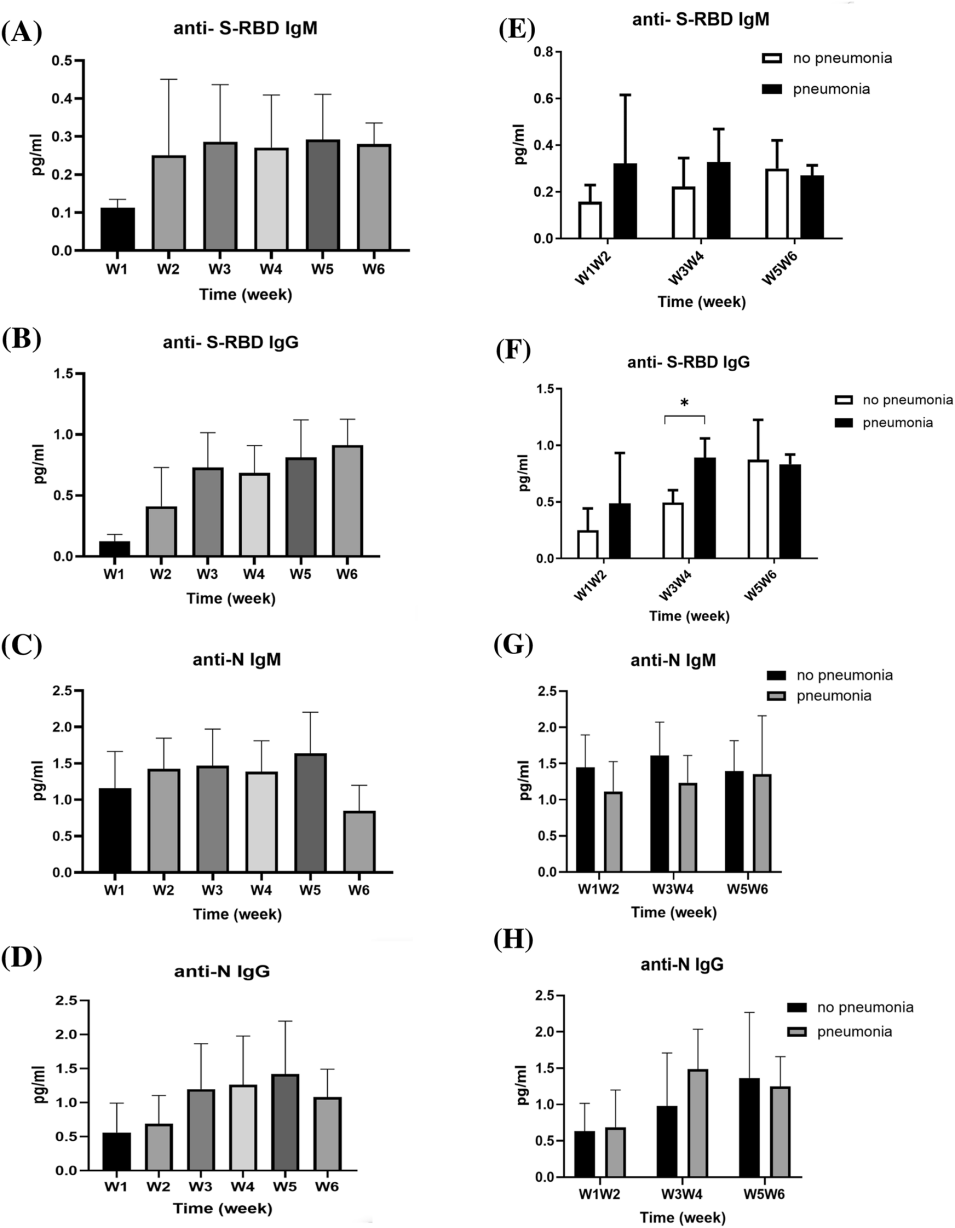

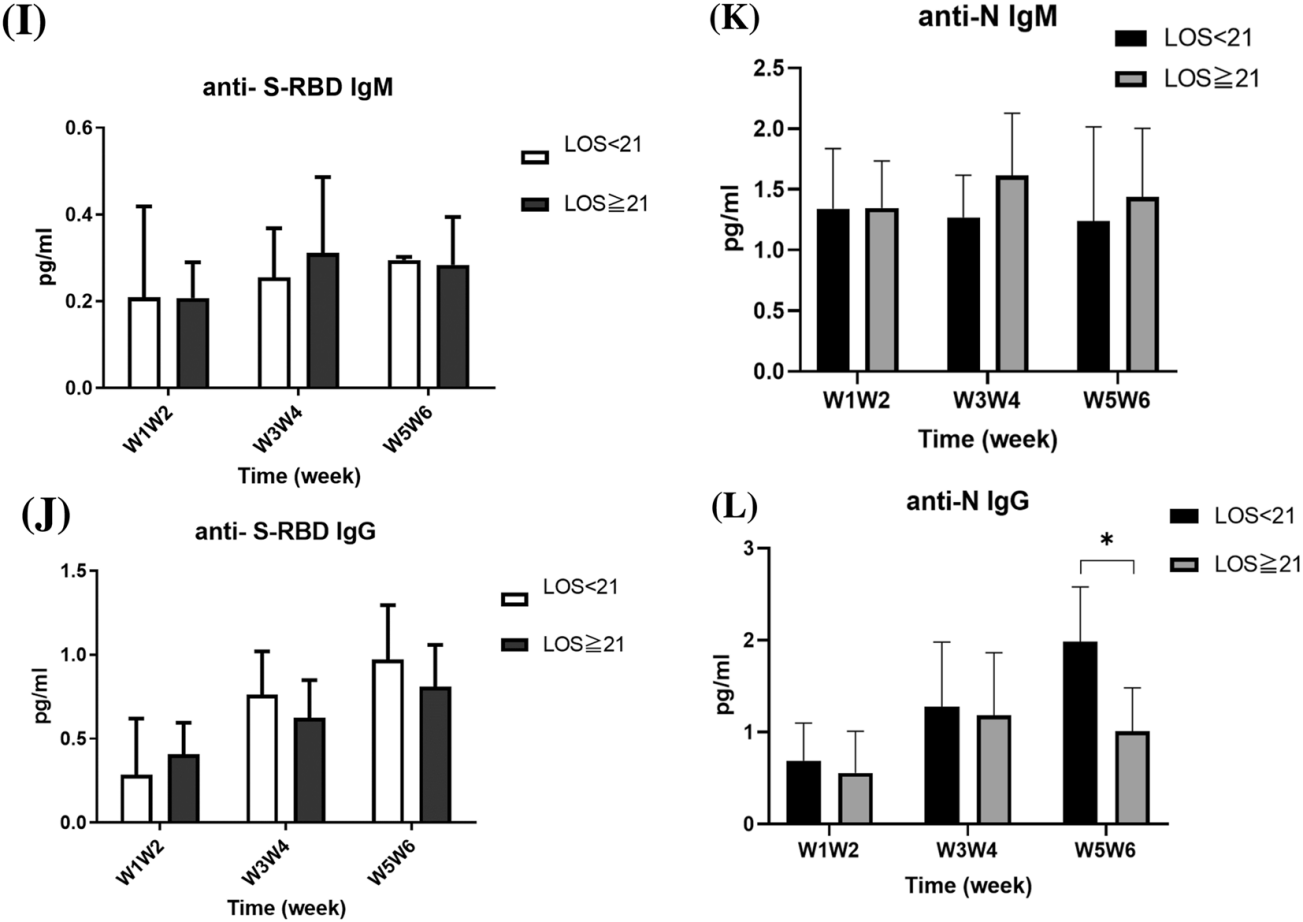
Serum anti-SARS-CoV-2 S-RBD IgM (**A**), IgG (**B**), anti-SARS-CoV-2 N protein IgM (**C**), and IgG (**D**) levels of patients with COVID-19 over weeks 1–6 after symptom onset. These patients were subgrouped based on pneumonia diagnosis. The anti-SARS-CoV-2 S-RBD IgM (**E**), IgG (**F**), anti-SARS-CoV-2 N protein IgM (**G**), and IgG (**H**) levels of the subgrouped patients were determined. The patients were also subgrouped based on the duration of hospitalization (≥ 21 d vs. < 21 d). The anti-SARS-CoV-2 S-RBD IgM (**I**), IgG (**J**), anti-SARS-CoV-2 N protein IgM (**K**), and IgG (**L**) levels of the subgrouped patients are shown. *p < 0.05

The levels of anti-SARS-CoV-2 S-RBD IgM and IgG, as well as anti-N IgM and IgG, in patients were measured over weeks 1–6 after symptom onset, and the results are presented in Fig. 4. The anti-S IgM levels increased in the second week after symptom onset and stabilized in the following 4 weeks (Fig. 4A). The anti-S IgG levels also increased in the second week after symptom onset and continued to increase in the following 4 weeks (Fig. 4B). Conversely, the anti-N IgM levels increased in the first week after symptom onset and decreased in week 6 (Fig. 4C), while the anti-N IgG levels increased in the third week after symptom onset (Fig. 4D). Starting from week three and four after symptoms onset, patients with pneumonia displayed significantly higher anti-S IgG titers compared with those without pneumonia (0.893 ± 0.168 vs. 0.495 ± 0.110 pg/mL; *p* < 0.0001) (Fig. 4F). In patients with varying lengths of hospitalization, starting from week five and six after symptoms onset, those with shorter hospitalization duration displayed significantly higher anti-N IgG titers compared with patients with longer hospitalization duration (1.982 ± 0.597 vs. 1.012 ± 0.468 pg/mL; *p* = 0.002) (Fig. 4L).

**Fig. 5:**
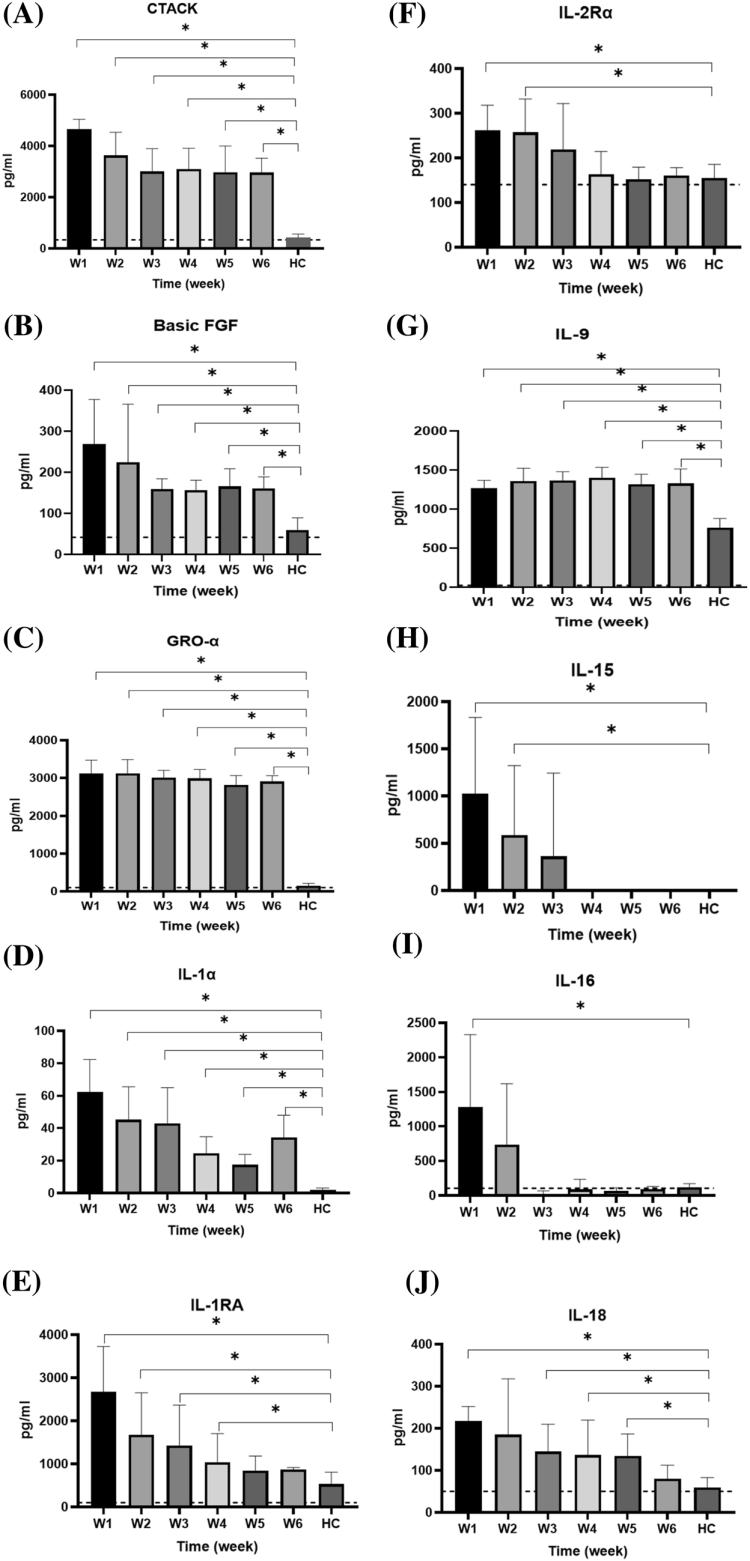

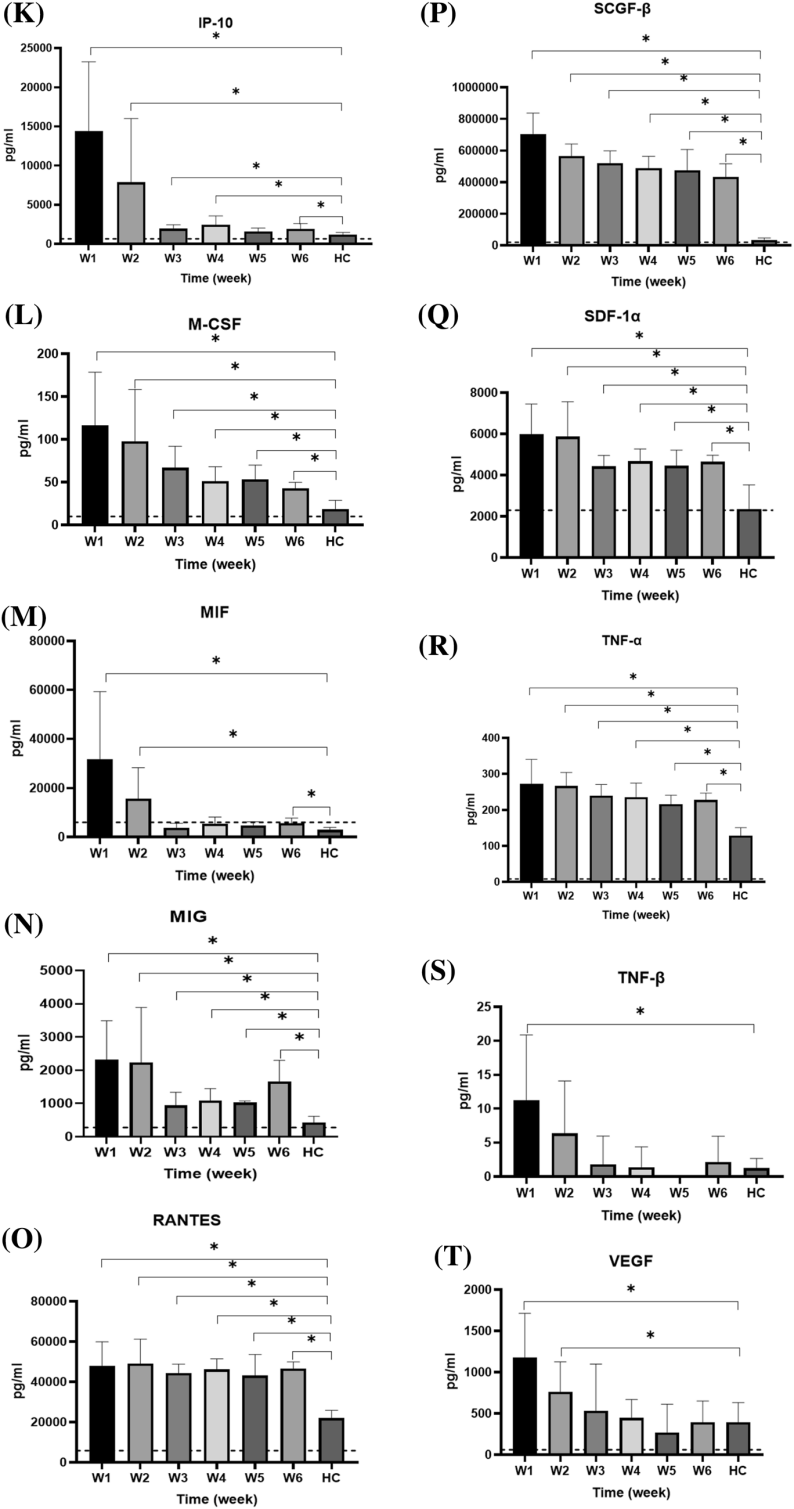

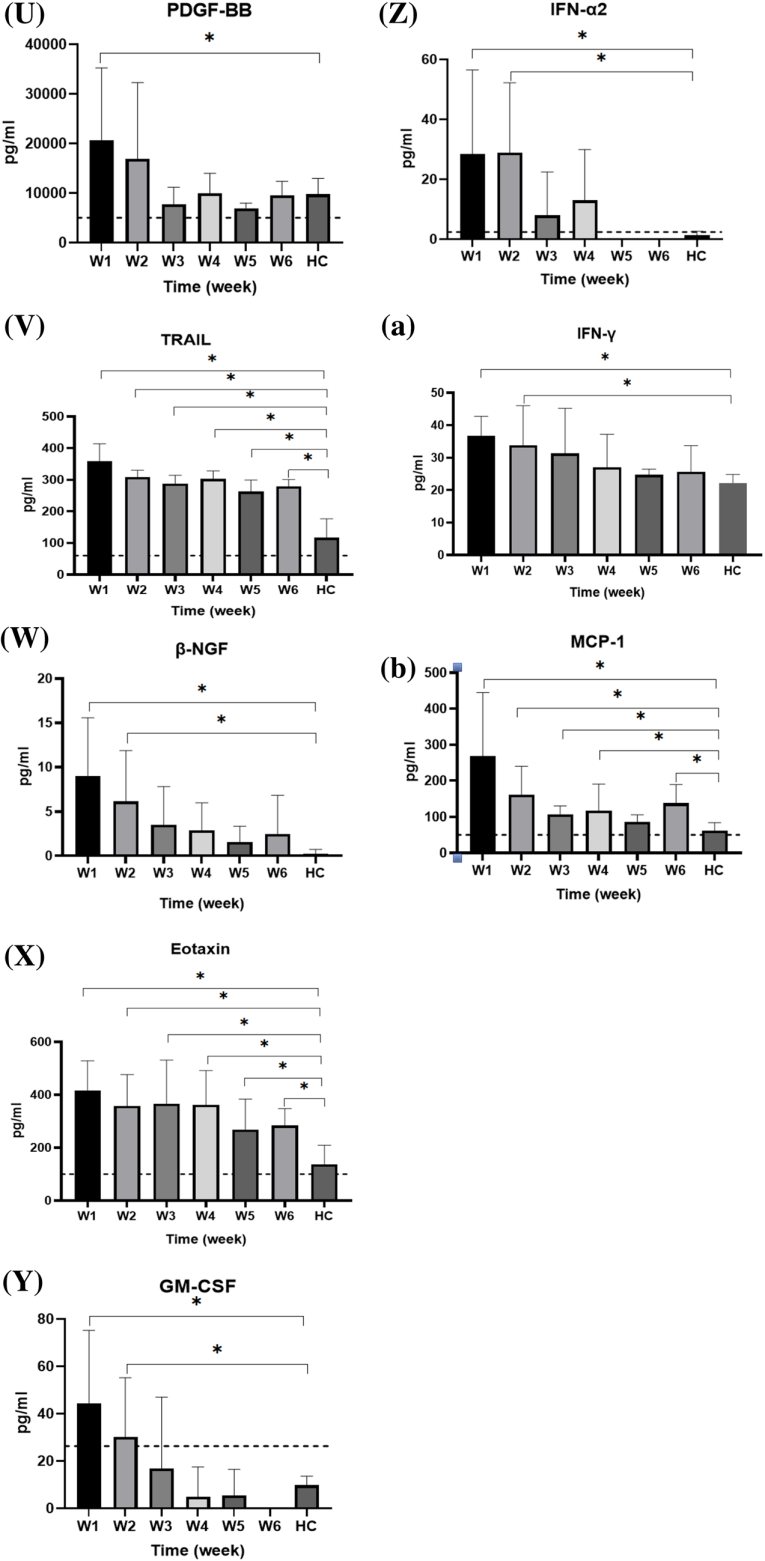
Serum cytokine levels in healthy controls (HC) and patients with COVID-19 over weeks 1–6 after symptom onset. The levels of each cytokine in healthy subjects, according to previous literature, are indicated as dotted lines. The serum cytokine levels, collected weekly from patients starting from symptom onset, were measured and compared with the serum cytokine levels in healthy controls using an independent *t-*test. *p < 0.05

Serum cytokines that increased between weeks 1 and 6 after symptom onset are shown in Fig. 5, while those that did not increase are shown in Supplementary Fig. S1. Serum CTACK, basic FGF, GRO-α, IL-1α, IL-1RA, IL-2Rα, IL-9, IL-15, IL-16, IL-18, IP-10, M-CSF, MIF, MIG, RANTES, SCGF-β, SDF-1α, TNF-α, TNF-β, VEGF, PDGF-BB, TRAIL, β-NGF, eotaxin, GM-CSF, IFN-α2, INF-γ, and MCP-1 in the enrolled patients were significant higher than those in healthy subjects during the first week after symptom onset. CTACK, basic FGF, IL-1α, IL-1RA, IL-2Rα, IL-15, IL-16, IL-18, IP-10, M-CSF, MIF, MIG, SCGF-β, SDF-1α, TNF-α, TNF-β, VEGF, PDGF-BB, TRAIL, β-NGF, eotaxin, GM-CSF, IFN-α2, INF-γ, and MCP-1 levels gradually decreased in the following weeks. Furthermore, IL-15, IL-16, MIF, TNF-β, VEGF, PDGF-BB, and GM-CSF levels returned to the normal range around the third week after symptoms onset.

### Serum cytokine levels comparison between patients with and without pneumonia

**Fig. 6:**
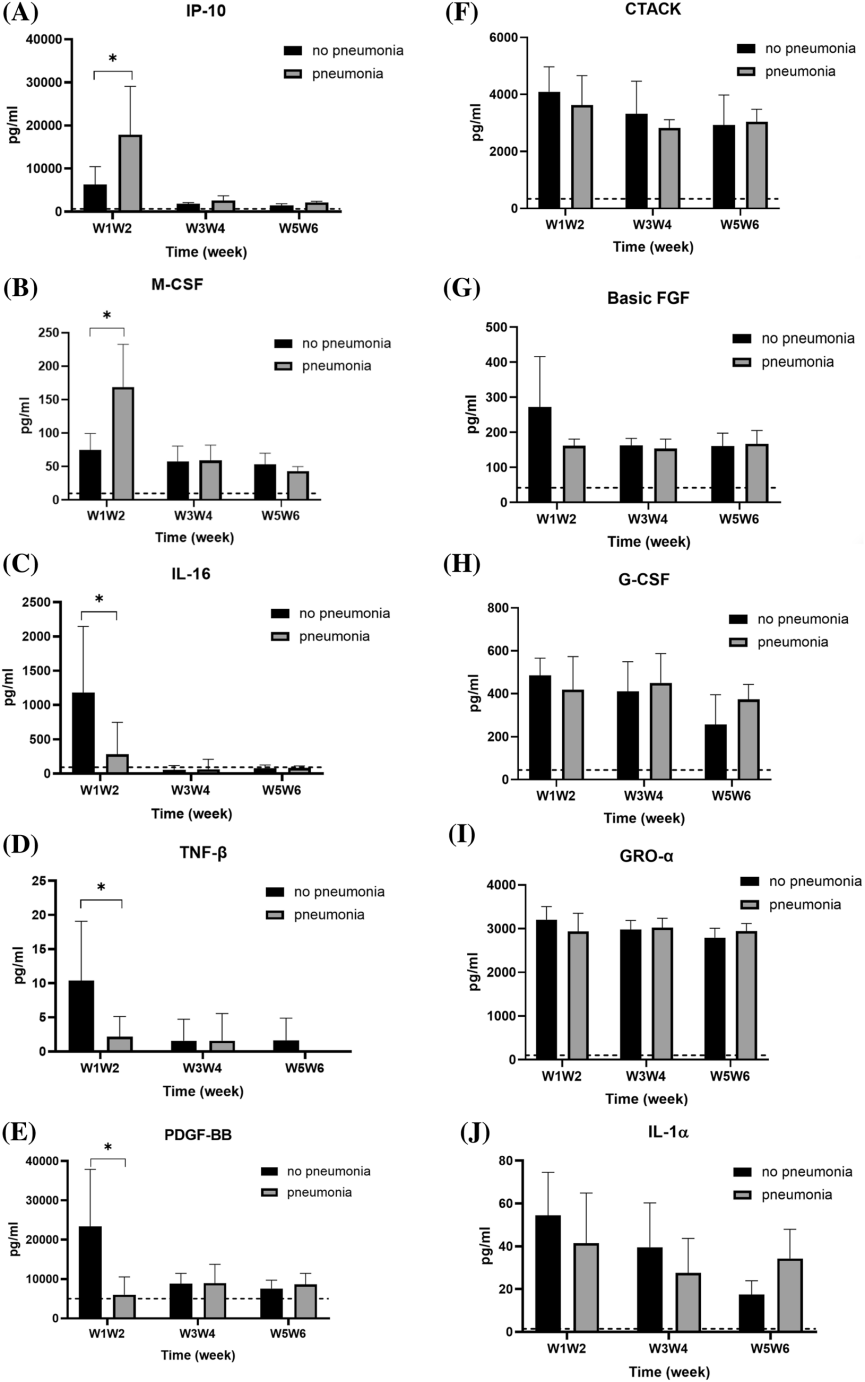

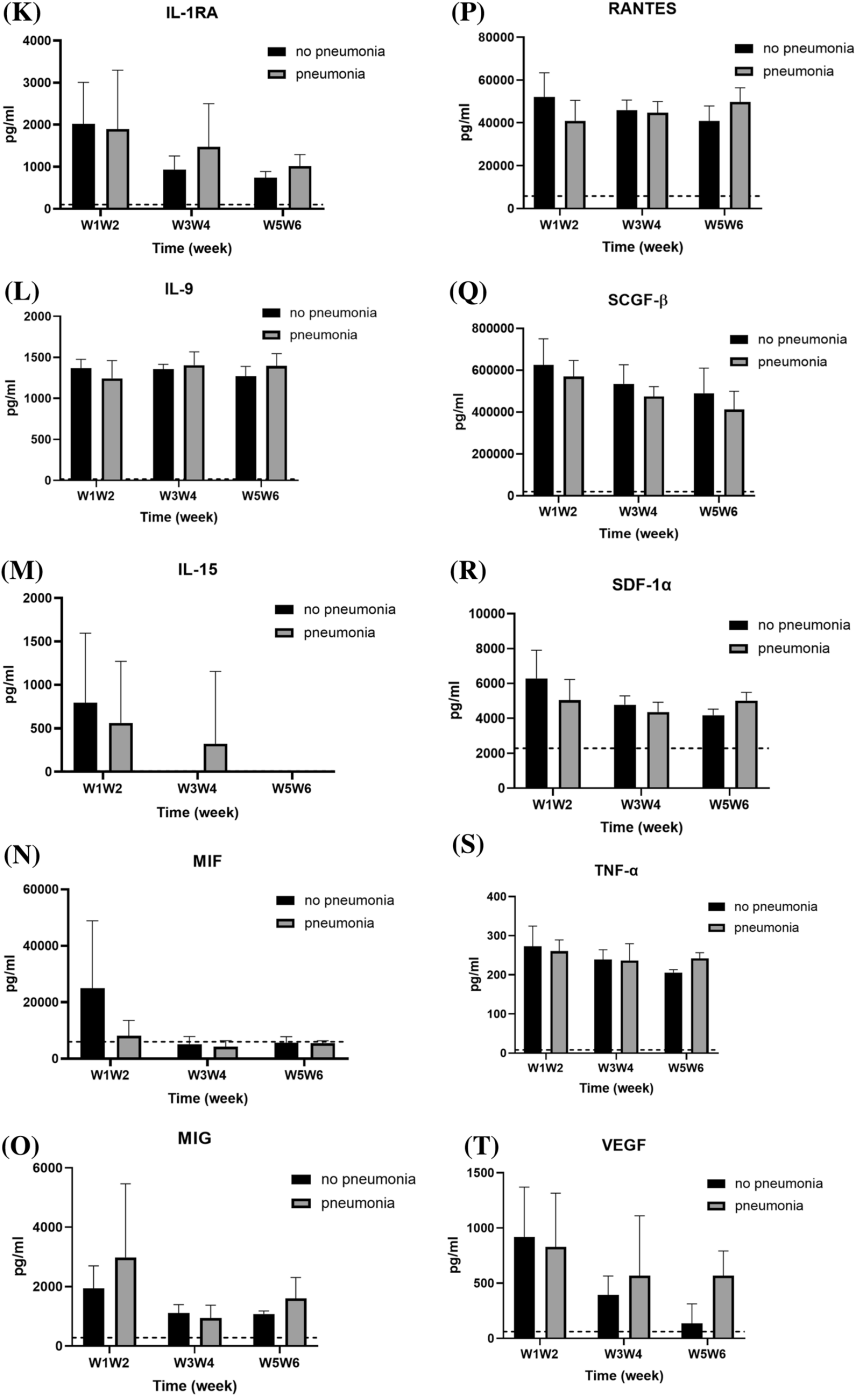
Comparison of serum cytokine levels of COVID-19 patients subgrouped based on pneumonia diagnosis. The levels of each cytokine in healthy subjects, according to previous literature, are indicated as dotted lines. *p < 0.05

The enrolled patients were further classified based on the presence or absence of pneumonia; comparison of serum cytokine levels between the two groups is shown in Fig. 6. Patients with pneumonia expressed significantly higher levels of IP-10 (17,875.20 ± 11,214.72 vs. 6304.32 ± 4145.05 pg/mL; *p* = 0.017) and M-CSF (168.76 ± 63.71 vs. 74.68 ± 24.64 pg/mL; *p* = 0.002) in comparison with those without pneumonia (Fig. 6A, B) and lower levels of IL-16 (281.87 ± 467.76 vs. 1178.46 ± 967.96 pg/mL; *p* = 0.046), TNF-β (2.17 ± 2.94 vs. 10.40 ± 8.69 pg/mL; *p* = 0.028), and PDGF-BB (6017.92 ± 4543.76 vs. 23,392.56 ± 14,481.68 pg/mL; *p* = 0.042) (Fig. 6C–E), especially during the first 2 weeks after symptoms onset. The serum levels of other tested cytokines did not significantly differ between these two groups.

### Effect of length of hospitalization on serum cytokine levels

**Fig. 7:**
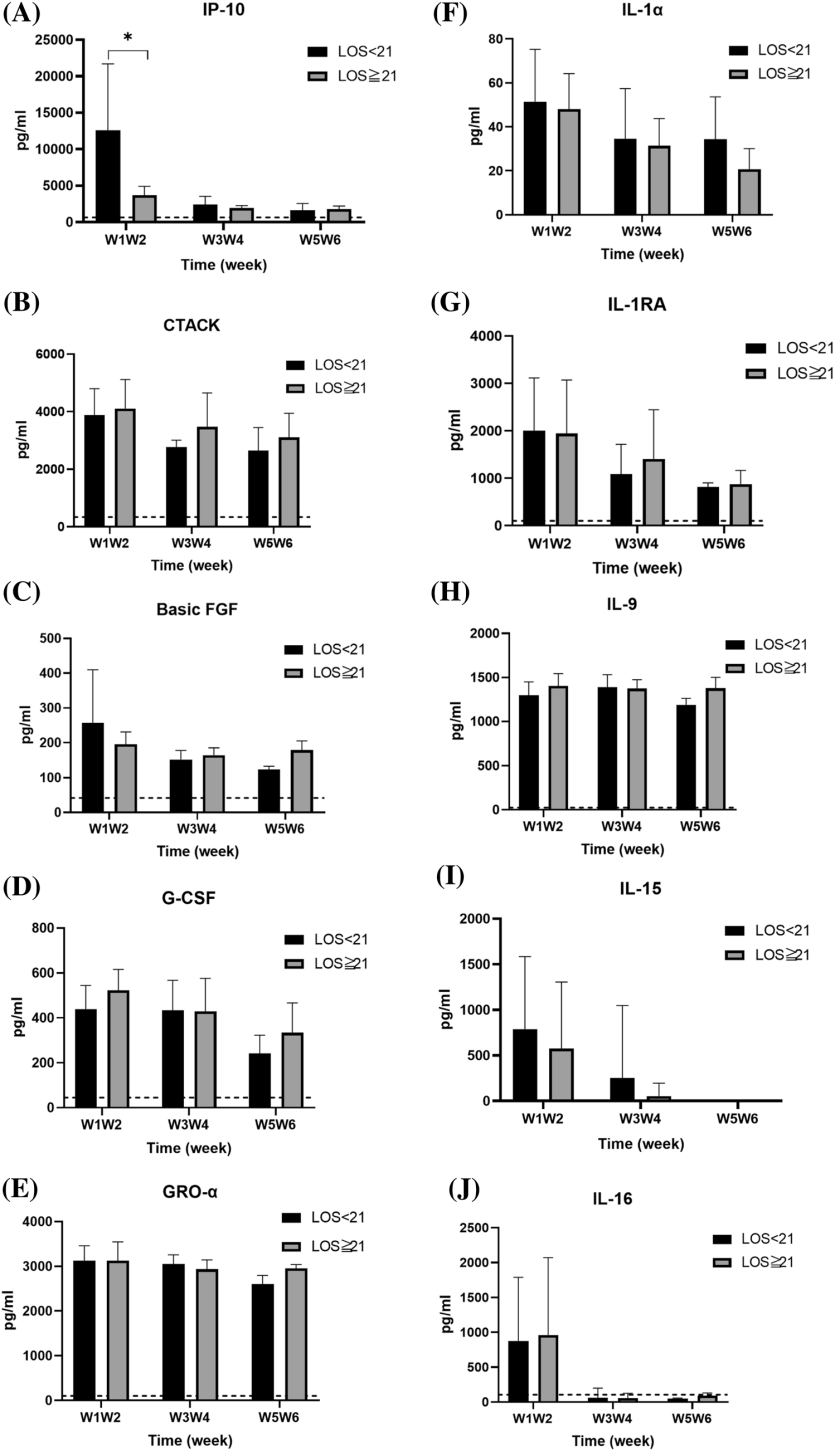

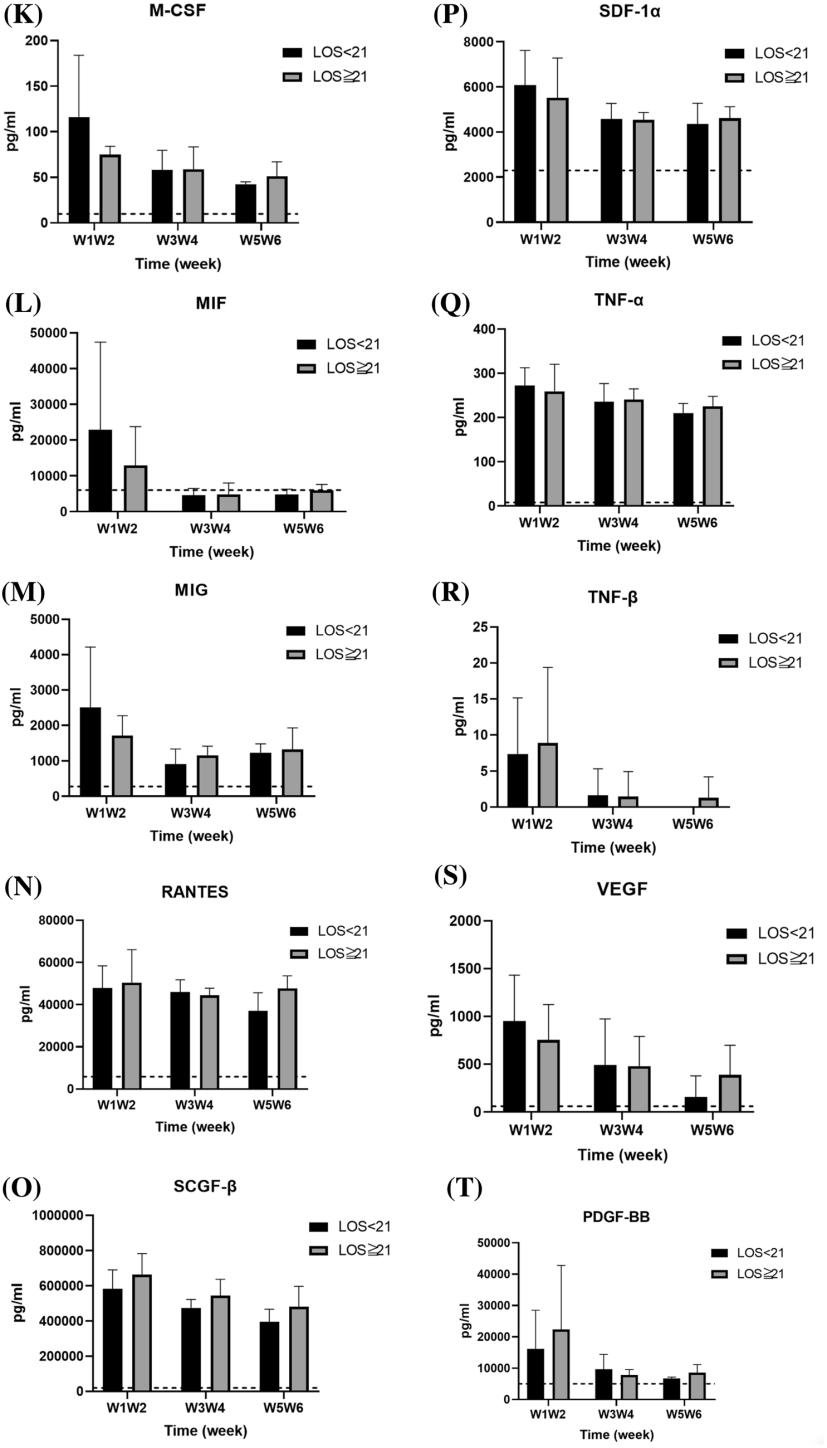
Comparison of serum cytokine levels of COVID-19 patients subgrouped based on the length of hospital stay (LOS) (≥ 21 d vs. < 21 d). The levels of each cytokine in healthy subjects, according to previous literature, are indicated as dotted lines. *p < 0.05

The enrolled patients were further classified based on the length of hospitalization (hospitalization ≥ 21 d vs. hospitalization < 21 d); comparison of their serum cytokine levels is shown in Fig. 7. Patients with a shorter duration of hospitalization showed higher serum IP-10 levels (12,614.76 ± 9081.56 vs. 3676.74 ± 1224.56 pg/mL; *p* = 0.019) than those with a longer duration of hospitalization (Fig. 7A). The serum levels of other tested cytokines did not significantly differ between the two groups.

## Discussion

SARS-CoV-2 can induce a life-threatening systemic inflammatory state by triggering various cytokines, chemokines, and hyperactive immune cells. Our study revealed that the N protein, rather than the S protein, of SARS-CoV-2 can trigger lung epithelial A549 cells to express various cytokines, namely, IP-10, RANTES, IL-16, MIP-1α, basic FGF, eotaxin, IL-15, PDGF-BB, TRAIL, VEGF-A, and IL-5. These may initiate subsequent cytokine cascades and promote systemic inflammatory syndromes. We also found that patients with COVID-19 showed significantly high levels of serum CTACK, basic FGF, GRO-α, IL-1α, IL-1RA, IL-2Rα, IL-9, IL-15, IL-16, IL-18, IP-10, M-CSF, MIF, MIG, RANTES, SCGF-β, SDF-1α, TNF-α, TNF-β, VEGF, PDGF-BB, TRAIL, β-NGF, eotaxin, GM-CSF, IFN-α2, INF-γ, and MCP-1. High serum IP-10 and M-CSF levels accompanied by low IL-16, TNF-β, and PDGF-BB levels may be associated with the development of pneumonia. In contrast, high serum IP-10 levels may be associated with rapid virus elimination in patients with COVID-19.

In our study, the lung epithelial A549 cells displayed a greater immune response to N-Bac compared with that of monocytic THP-1 cells. This finding suggests that cytokine cascades initiate in the lung epithelial cells and not in the circulating monocytes of patients with COVID-19, which may explain the occurrence of severe COVID-19 in patients with pneumonia and milder forms in those without pneumonia. IP-10, which recruits activated T helper 1 (Th1) cells for host defense against lung intracellular pathogens, and IL-16, a lymphocyte chemoattractant factor, are released by both bronchial and alveolar epithelial cells [14, 15]. The blockade of eotaxin and IL-16 causes 70% inhibition of eosinophil chemotactic activity to the lung in pulmonary disorders [16]. Collectively, these studies suggest that bronchial and alveolar epithelial cells secrete cytokines, such as IP-10, IL-16, or eotaxin, leading to subsequent pulmonary disorders, consistent with our in vitro experiments.

Stimulation of THP-1 cells with S-bac or N-bac increased the expression levels of CD-86 and IL-6 in THP-1 cells, while those of CD-204 and TGF-β remained unchanged, suggesting that both S-Bac and N-Bac stimulations induce THP-1 cells toward M1 polarization. Most COVID-19 studies have focused on the role of the S protein of SARS-CoV-2 in COVID-19 pathoetiology. Previous in silico studies predicted that cell surface Toll-like receptors (TLRs), especially Toll-like receptor 4 (TLR4), are likely to be involved in recognizing molecular patterns, probably SARS-CoV-2 S protein, to induce inflammatory responses [17, 18]. Another study found that the S protein can activate TLR4 and induce IL-1β production in THP-1 cells [19]. These studies only suggest that the S protein may induce IL-1β production by activating TLR4 in macrophages. Whether S protein-induced IL-1β production represents the large proportion of whole IL-1β production in macrophages and IL-1β production affects cytokine activation in COVID-19 patients remains unknown. Moreover, the SARS-CoV-2 S protein can only prime inflammasome formation and release of mature IL-1β in macrophages derived from patients with COVID-19 but not in macrophages from healthy SARS-CoV-2 naive individuals [20]. Therefore, IL-1β may not be a key cytokine in the pathoetiology of patients first time contracting COVID-19. The N protein interacts directly with the Nucleotide-binding oligomerization domain leucine-rich repeat, and pyrin domain containing 3 (NLRP3) inflammasome can promote IL-1β and IL-6 activation and cause subsequent lung injury in mouse models [21]. Furthermore, the N protein can promote the activation of Nuclear factor κB (NF-κB) signaling by enhancing the association between TGF-beta-activated kinase 1 and IκB kinase complex [22] and function as a pathogen-associated molecular pattern to directly bind to TLR2 and activate NF-κB and mitogen-activated protein kinase signaling in endothelial cells [23]. Therefore, the N protein can induce pro-inflammatory cytokines through promoting the activation of NF-κB signaling and NLRP3 inflammasome. However, these studies could not determine which protein is the important virulence factor in cytokine activation and subsequent lung injury. In contrary, our study revealed that N protein, rather than S protein, can trigger A549 cells to express considerably high levels of various cytokines to promote a cytokine storm. Moreover, S protein is a leading target antigen in the development of COVID-19 vaccine; however, nonsynonymous mutations developed in the S protein could create SARS-CoV-2 variants as the epidemic progressed [24, 25]. These variants reduced the effectiveness of current S protein recombinant vaccines and contributed to the continuation of the COVID-19 pandemic. In contrast, the N gene is more conserved and stable, with 90% amino acid homology and fewer mutations over time [26–29], hence is an appropriate target in the development of new generation medicine or COVID-19 vaccines. Due to the conserved nature of the N protein, despite the continuous emergence of new variants of SARS-CoV-2, the results of our in vitro study may still be applicable to other strains.

Taiwan has been able to contain the pandemic at the time of study. Most of our enrolled patients were classified as imported cases (*n* = 25; 92.59%) and had gone abroad for education or tourism; most of them had relatively mild illnesses. Besides, in Taiwan at that time, all confirmed COVID-19 patients could only stop quarantine after three negative COVID-19 tests. Due to the long communicability period of COVID-19 and the relatively mild disease severity of confirmed patients in our hospital, all our patients recovered before showing three negative COVID-19 tests. Therefore, the length of quarantine in our study corresponded to the time needed for virus eradication rather than disease severity.

Patients with pneumonia expressed a considerably higher titer of anti-SARS-CoV-2 S-RBD IgG than those without. In previous studies, anti-S IgM and IgG titers remarkably correlated with the viral load and disease severity in patients with COVID-19 [30, 31]. The anti-S antibody response developed considerably faster with higher titers in patients who eventually died of SARS [32]. The anti-S IgG antibody-activated inflammatory macrophages and cytokines, such as MCP-1 and IL-8, cause severe lung injury in SARS-CoV-2-infected macaques [33]. Collectively, these studies suggest that increased anti-S IgG production may correlate with a robust inflammatory response and cause severe pulmonary injury in SARS-CoV-2 infection. Furthermore, patients with shorter hospitalization duration in our study had significantly higher anti-N IgG levels than the remaining patients. Previous studies found that SARS-CoV-2 N protein was highly immunogenic [34, 35], indicating that anti-N IgG may play a role in eliminating SARS-CoV-2 in patients with COVID-19.

IP-10 may play an important role in initiating cytokine cascades and final cytokine expression in patients with COVID-19. The MIG and IP-10/CXCR3 axis plays a crucial role in recruiting various immune cells, including T lymphocytes, natural killer cells, and macrophages, to damaged or inflamed tissues [36, 37]. The immune cell population in the lungs of patients with COVID-19 comprises a considerable proportion of T cells and monocytes, compared with patients with primary pneumonia infection without COVID-19 [38]. While T cells and monocytes are relatively rare in healthy lungs [39], their accumulation is assumed to be recruited by locally produced chemoattractant proteins, such as IP-10 and MIG. Besides, higher serum IP-10 levels are associated with a higher risk of severe Mycoplasma pneumoniae pneumonia in children [40] and higher risk of death in patients with ARDS [41]. These findings suggest that IP-10 is prone to development of pneumonia and tissue damage during inflammation. Moreover, in a neuroadapted John Howard Mueller strain of mouse hepatitis virus, IP-10 is responsible for viral suppression after central nervous system inoculation [42]. Collectively, increased expression of IP-10 from lung epithelial cells recruited immune cells, including T lymphocytes, thereby exacerbating immune reaction and organ damage, causing severe pneumonia, and eliminating viral loads, resulting in a shorter time of viral shedding.

In our study, high serum M-CSF levels were associated with the development of pneumonia in patients with COVID-19. M-CSF is a necessary growth factor for recruiting and expanding lung monocytes. It also leads to the transition of monocytes to macrophages during infection [43, 44] and contributes to tissue repair during inflammation [44]. Macrophages differentiated in the presence of M-CSF, adenosine, and PGE2 induced the downregulation of inflammatory mediators and upregulation of growth factors [44]. In contrast to IP-10, M-CSF levels were elevated in COVID-19 pneumonia patients, which reduced tissue damage during inflammation.

IL-1RA is a receptor for pro-inflammatory cytokines, specific to the activity of both IL-1α and IL-1β [45]. The blockade of IL-1 in patients with COVID-19 considerably improved survival and shortened hospital stay [46]. However, treatment with canakinumab, an IL-1β receptor inhibitor, did not reduce the need for intermittent mandatory ventilation or mortality of patients with COVID-19 [47]. These findings suggest that IL-1RA and IL-1α, rather than IL-1β, may play a role in SARS-CoV-2 infection, consistent with our findings.

The expression of vascular endothelial growth factor (VEGF) was also increased in our study patients and lung epithelial A549 cells triggered by SARS-CoV-2 N protein. In a previous clinical trial involving 26 patients with severe COVID-19, bevacizumab, an anti-VEGF neutralizing antibody, plus standard care improved the PaO2/FiO2 ratio after 24 h. By day 28, 92% patients demonstrate oxygen-support improvement, 65% patients were discharged, and none show worsen oxygen support or death [48, 49]. Collectively, these data suggest that VEGF-induced vascular changes, including angiogenesis, alteration of vascular permeability, and inflammation, may cause life-threatening defects in patients with severe COVID-19.

Dr. Yang et al. had reported that the expression levels of IP-10, MCP-3, HGF, MIG, and MIP-1α are significantly higher in critically ill patients, followed by severe and then the moderate patients [50]. The study conducted by Dr. Yang focused on the cytokines those may relate to the development of severe or critical diseases. Among the patients they enrolled, 22% were critical illness patients, 50% were severe disease patients, and 28% were moderated disease patients. No patients with mild disease were enrolled. Although serum levels of IP-10, MCP-3, HGF, MIG, and MIP-1α were all higher in critical and severe disease patients than healthy control, the serum levels of MCP-3, HGF, MIG, and MIP-1α were no difference between patients with moderate disease and healthy control. These were indicated that these cytokines were markers to predict severe and critical disease progression, but not moderate disease. On the contrary, patients enrolled in our study were relatively non-illness. Among them, 3.7% were critical patients, 14.8% were severe disease patients, 44.4% were moderate disease patients, and 37% were mild disease patients. Considering the substantial influence of lung epithelial cells on the initiation of cytokine cascades in our in vitro study, as well as the impact of pneumonia on the clinical outcomes of COVID-19 patients, the clinical segment of our study is crafted to investigate the correlation between cytokine expression and the development of pneumonia. Our findings revealed that serum levels of IP-10 and M-CSF were significantly elevated in patients with pneumonia (critical, severe, and moderate cases) compared with those without (mild cases).

Several cell membrane-based biomaterials derived from various types of cells have been developed. These membrane-based biomaterials, rich in biologically active proteins and phospholipids, are designed to treat inflammation, tumors, or autoimmune diseases by regulating immune cell function, exerting enzyme-like activity, or neutralizing cytokines [51, 52]. Applying this cell membrane-based biomaterials platform to the potential therapeutic targets found in our study, such as lung epithelial cells, N proteins, IP-10, or other cytokines, will help develop new treatment strategies to prevent the progression of severe COVID-19.

This study has some limitations. First, we used A549 human lung carcinoma epithelial cells, not primary lung epithelial cells to evaluate the cytokine response triggered by SARS-CoV-2 proteins. Primary cells are the gold standard for studying cell behavior in vitro. However, the utilization of primary cells may face obstacles, such as challenges associated with in vitro isolation and cultivation, and loss of phenotype over extended periods in culture. Human primary lung epithelial cells lose their phenotype and capacity over a period of 1–2 weeks when cultured in vitro [53, 54]. Cell lines are generally easier to cultivate compared with primary cells, exhibit a rapid proliferation rate and extended lifespan, and retain their phenotype when maintained in culture. Therefore, the human lung adenocarcinoma cell line A549 is extensively used in lung cell biology. However, despite exhibiting similar responses to viral infection compared to primary cells, A549 cells show limited cytokine response [55]. The suitability of using A549 cells in COVID-19 studies is also a matter of discussion due to the low levels of angiotensin-converting enzyme 2 (ACE2) expression [56]. However, although the A549 cells is less sensitive to SARS-CoV-2 infection, the expression of ACE2 in A549 cells has been well documented [56]. Indeed, several studies have successfully utilized A549 cells to assess the impact of SARS-CoV-2 infection [56–58]. Moreover, to gain better understanding of the cytokine storm and chronic autoimmune symptoms caused by SARS-CoV-2 infection, Dr. Wang et al. identified autoantigens from A549 cells that are strongly tied to diverse immune symptoms of COVID-19 [58]. By comparing the autoantigens they discovered in A549 cells with previously collected proteomic and transcriptomic data related to SARS-CoV-2 infection found in the Coronascape database, they found that out of the 348 autoantigen proteins identified in A549 cells, 291 of them (83.6%) had previously been documented as having changes in cells or patient tissues during SARS-CoV-2 infection in earlier scientific literatures. This finding suggests that, despite being less sensitive to SARS-CoV-2 infection when compared to primary lung epithelial cells, A549 cells are still appropriate for evaluating how cells respond to SARS-CoV-2 infection. Second, our study is a cross-sectional design study. Correlations between the expression of some cytokines and clinical characteristics of COVID-19 patients were found. However, correlation does not necessarily imply causation. Many confounding factors such as the rapidity of diagnosis, recognition of disease progression, and secondary complications both directly from COVID-19 and indirectly from its treatment might contribute to the observed associations. Further longitudinal studies remain warranted to provide stronger evidence regarding the relationship between cytokine levels and disease progression. Third, although we found that the N protein may trigger cytokine release in COVID-19 patients, the underlying molecular mechanisms driving this response were not explored; therefore, further investigations remain warranted.

In conclusion, our study suggested that the N protein of SARS-CoV-2 can preferentially trigger lung epithelial cells over macrophages to express IP-10 and other pro-inflammatory cytokines, thereby initiating cytokine cascades. In patients with COVID-19 and pneumonia, IP-10 may play a role in inflammation and virus elimination. Further studies are warranted to validate our findings.

### Supplementary Information

Below is the link to the electronic supplementary material.

**Supplementary file 1: Fig. S1**. Serum cytokines without elevation in patients with COVID-19 over weeks 1–6 after symptom onset. The levels of each cytokine in healthy subjects are indicated as dotted lines. (DOCX 1910 KB)

## Data Availability

The datasets used and/or analyzed during the current study are available from the corresponding author on reasonable request.
