# Supplementary material for: SARS-CoV-2 nucleocapsid protein, rather than spike protein, triggers a cytokine storm originating from lung epithelial cells in patients with COVID-19
Source: Infection. 2023 Dec 22;52(3):955–83. doi: 10.1007/s15010-023-02142-4 (PMC11143065; doi:10.1007/s15010-023-02142-4)
Supplement: Supplementary file 1 — Fig. S1. Serum cytokines without elevation in patients with COVID-19 over weeks 1–6 after symptom onset. The levels of each cytokine in healthy subjects are indicated as dotted lines. (DOCX 1910 KB) [file 15010_2023_2142_MOESM1_ESM.docx]

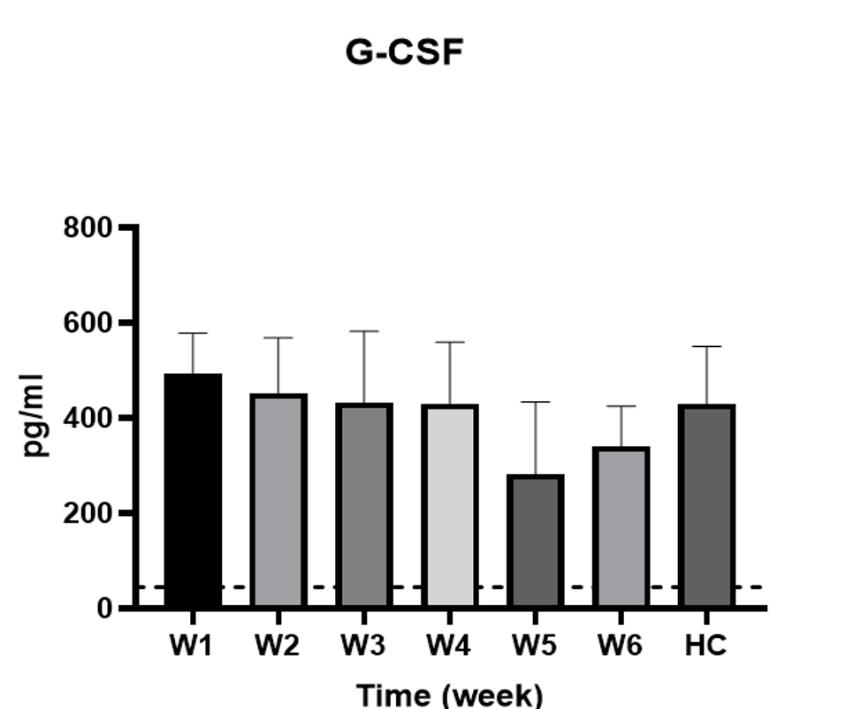


(A)


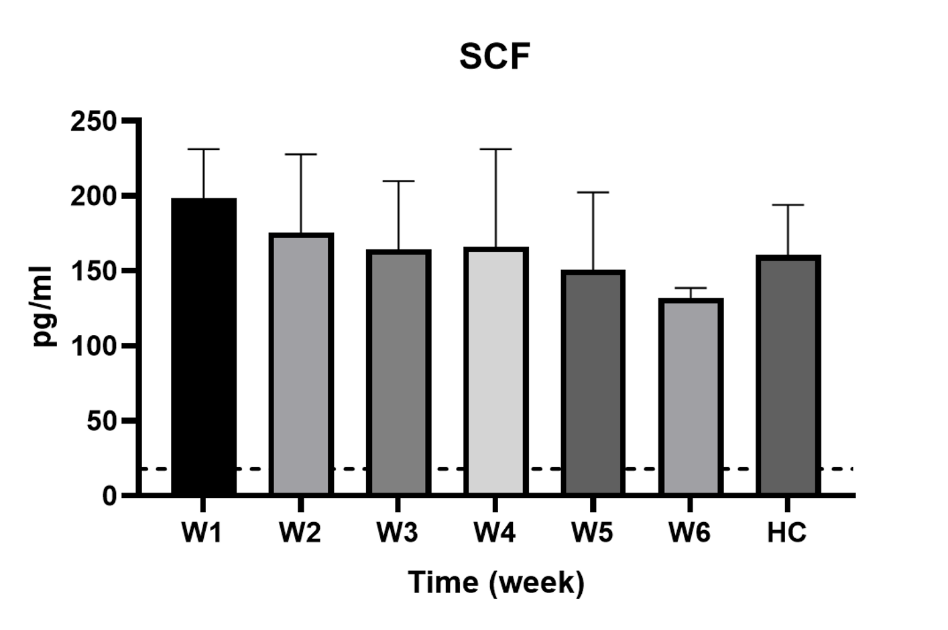


(B)


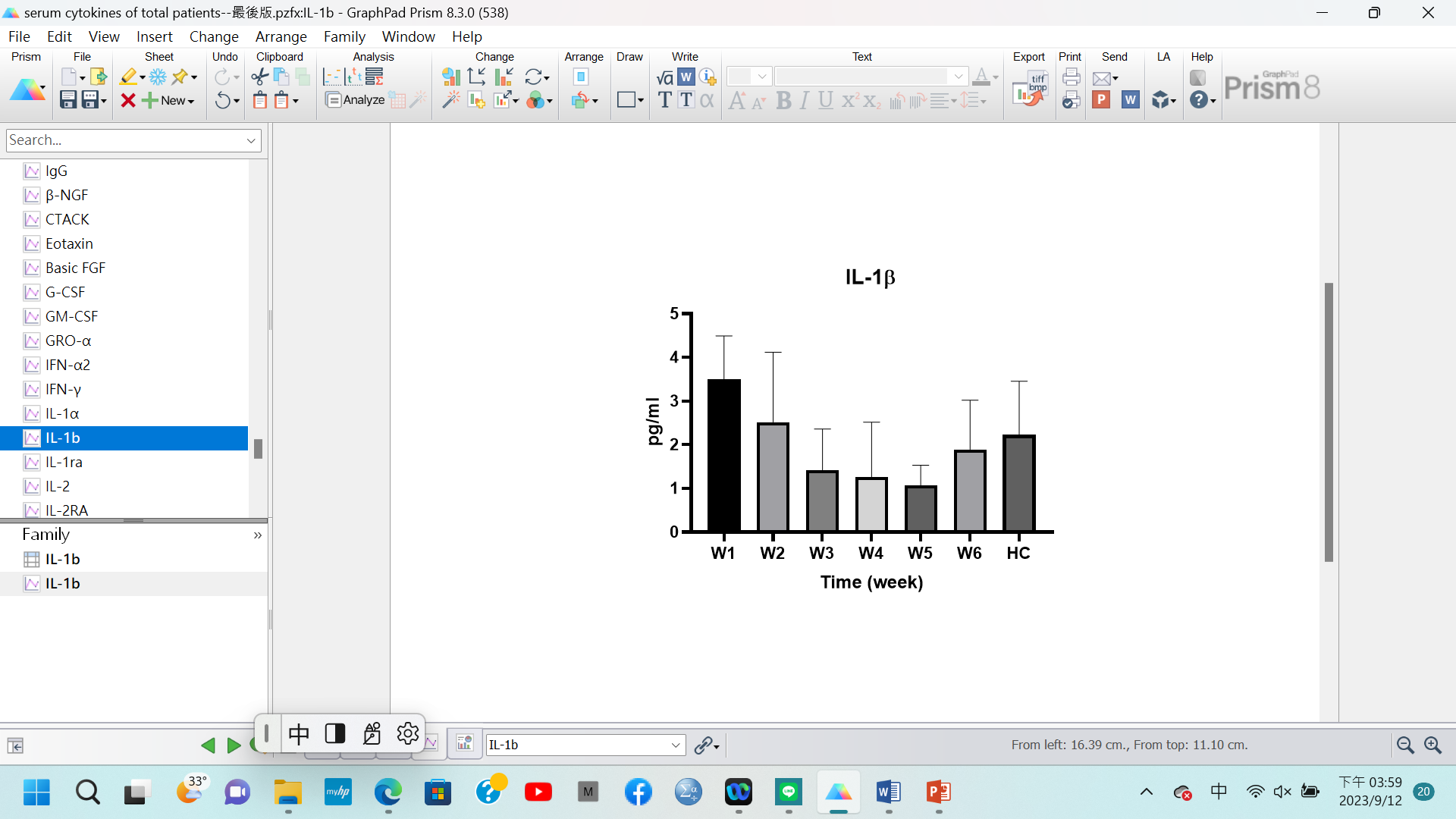


(C)


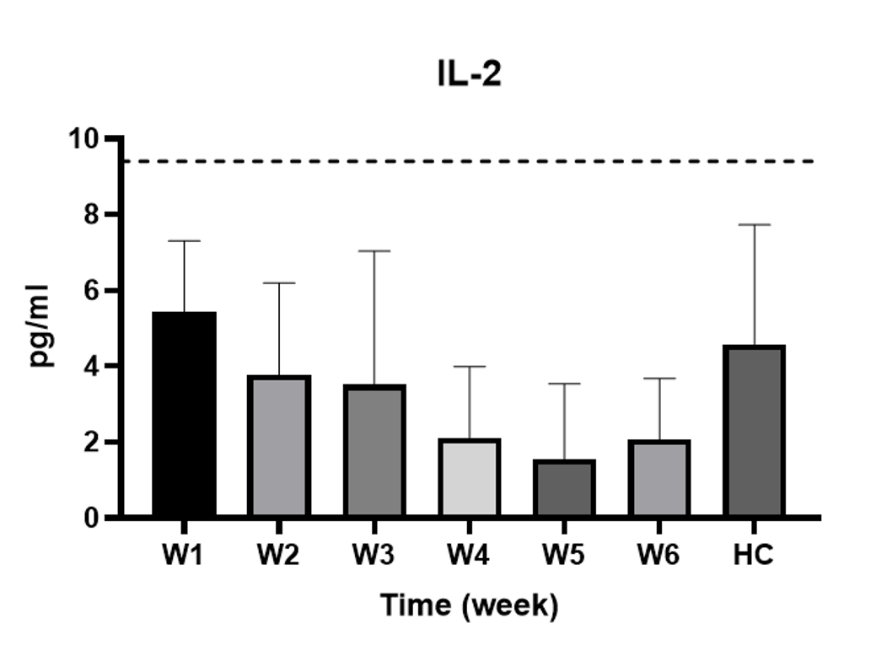


(D)


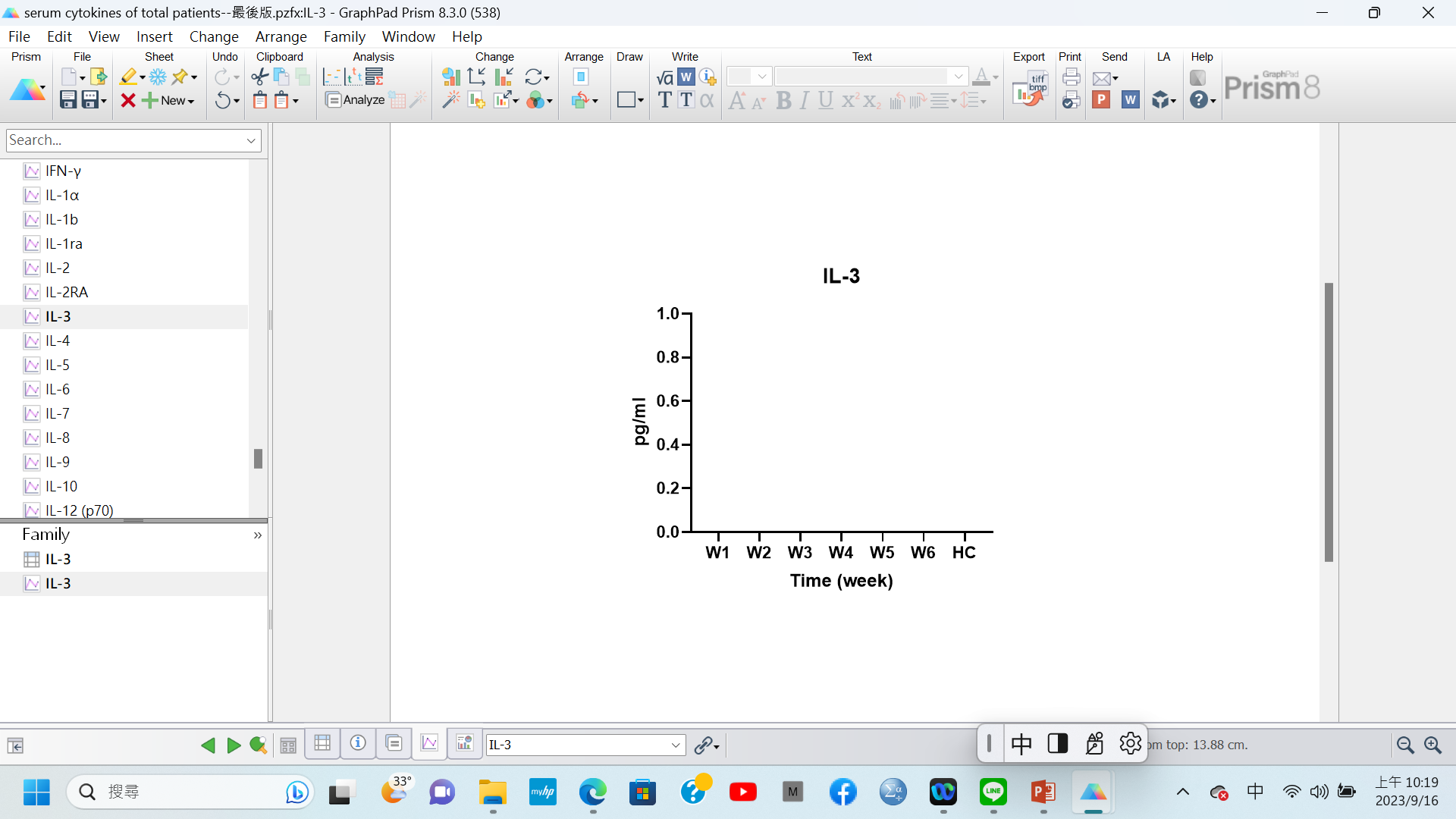


(E)


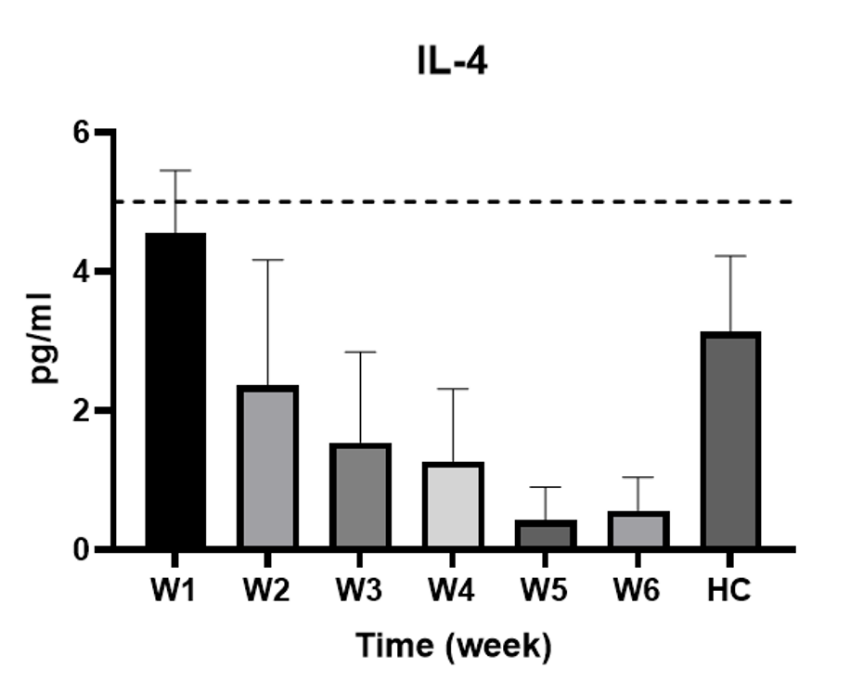


(F)


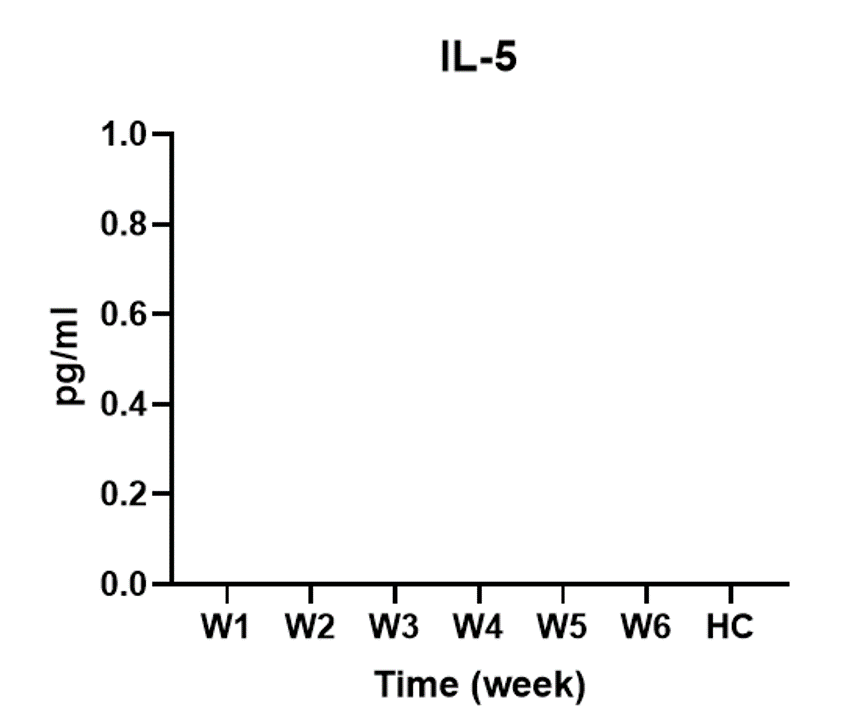


(G)


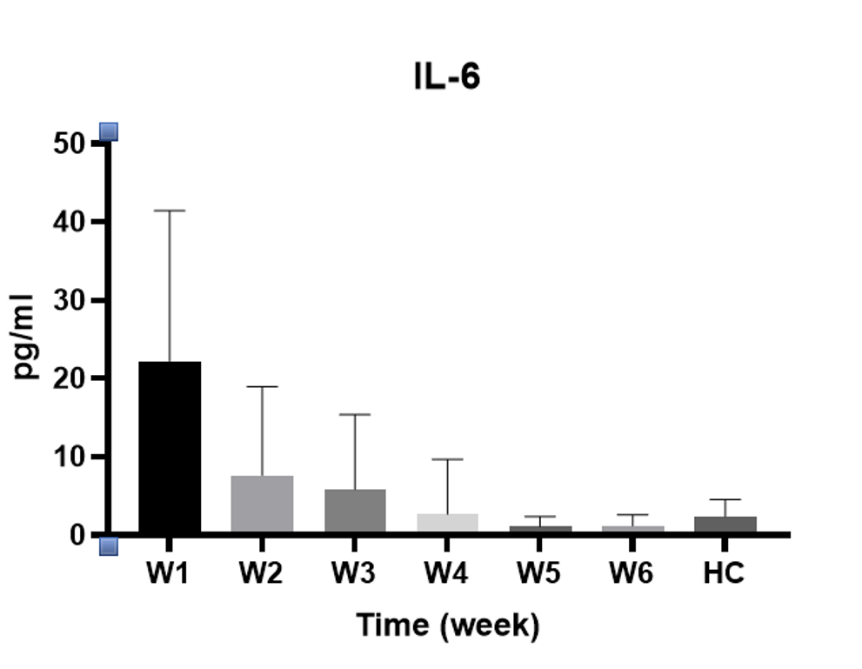


(H)


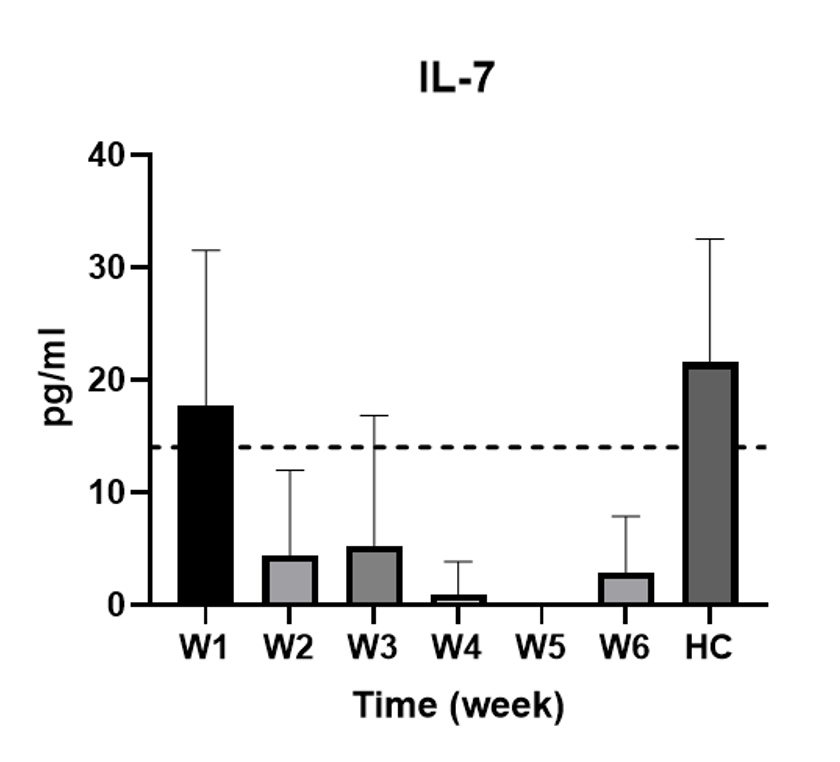


(I)


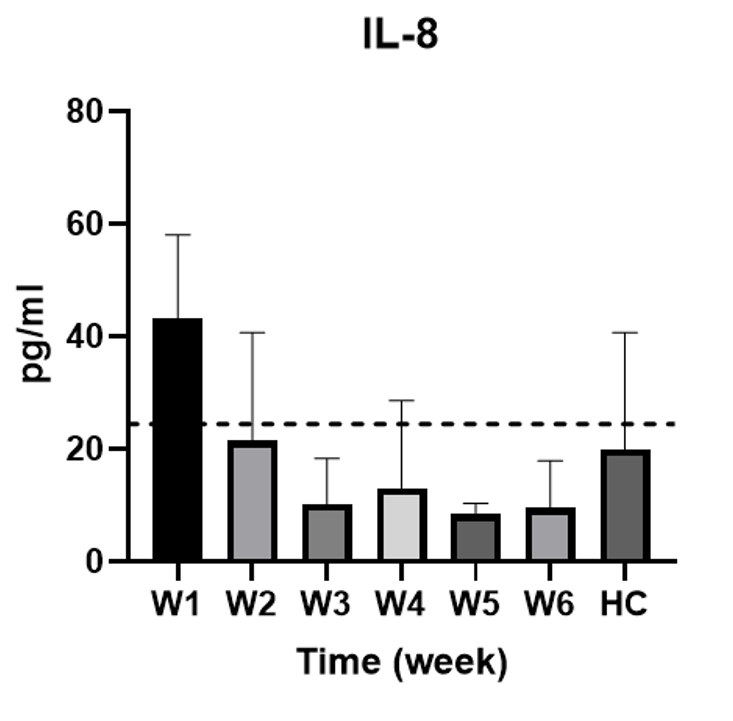


(J)


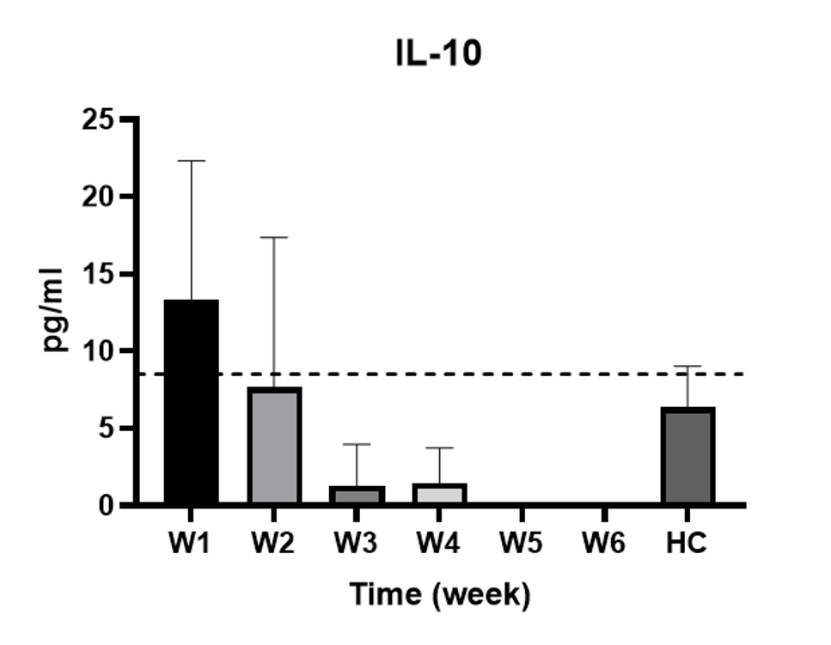


(K)


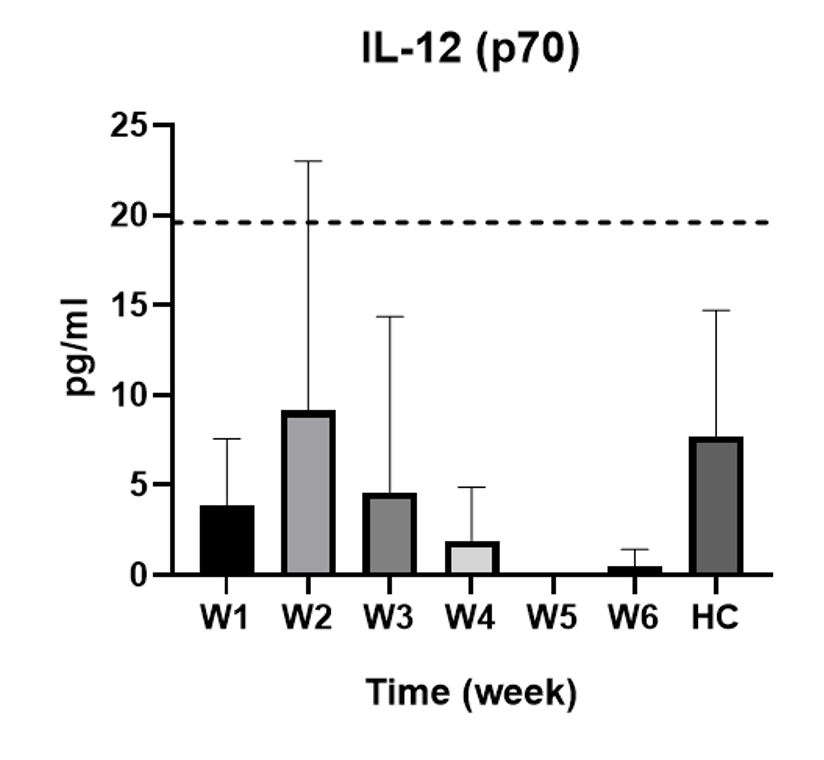


(L)


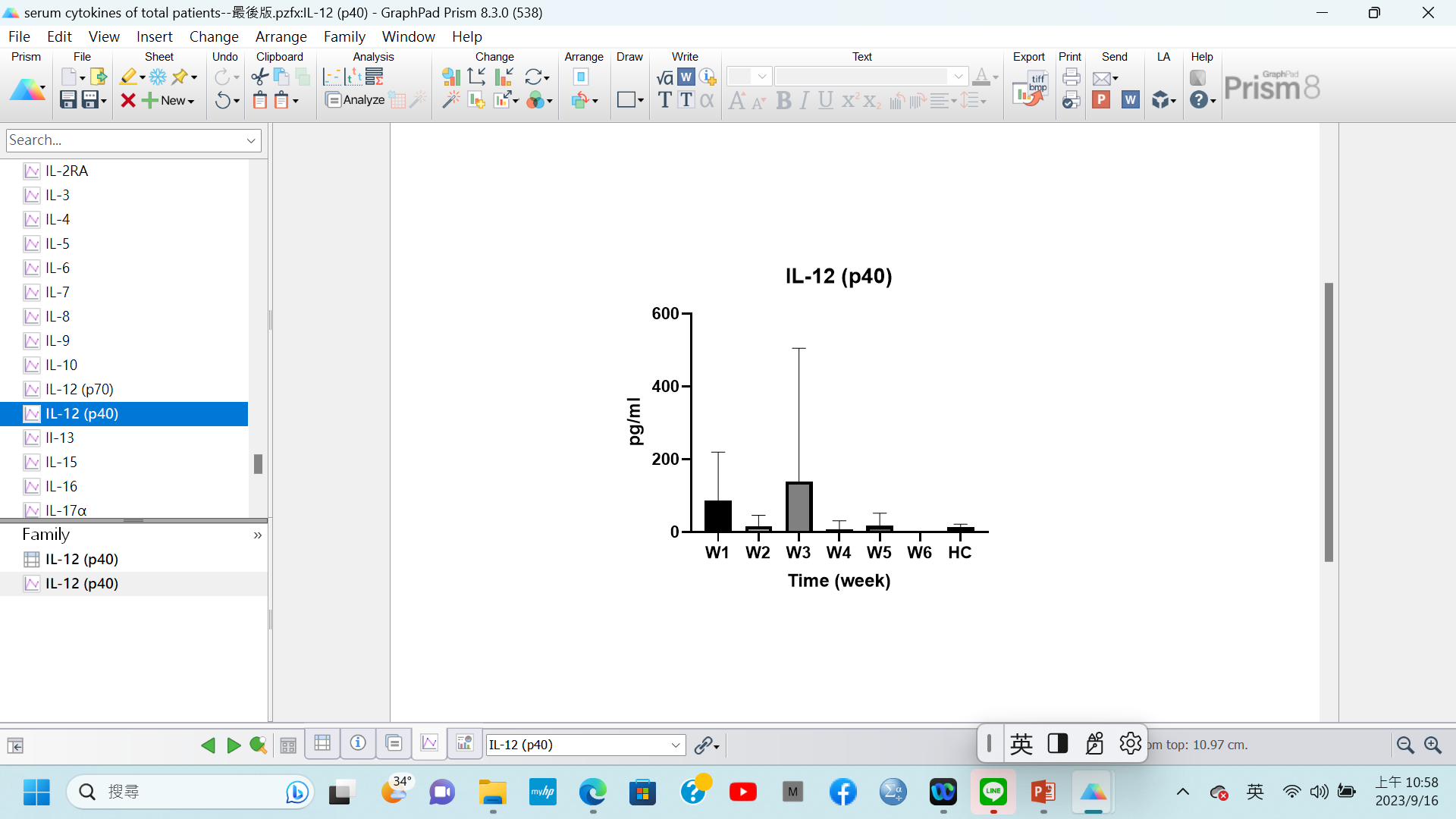


(M)


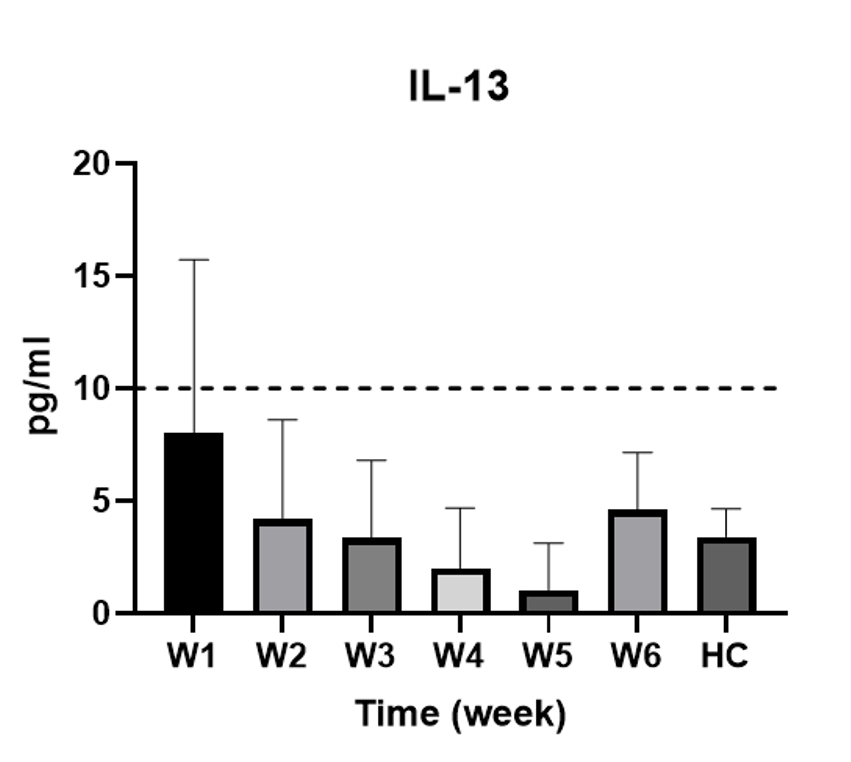


(N)


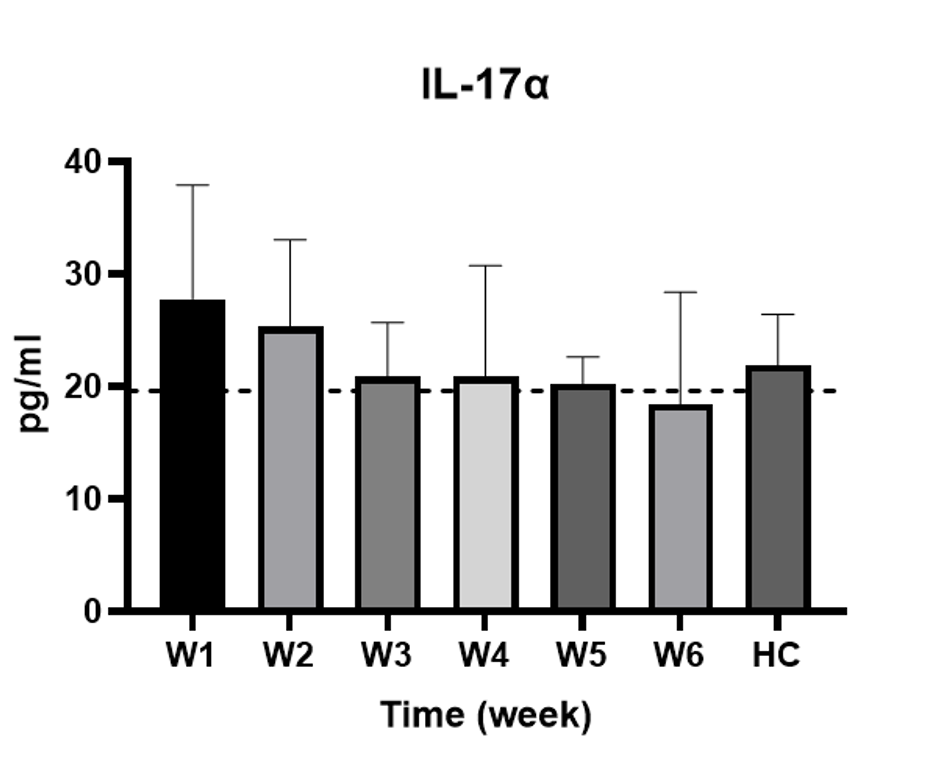


(O)


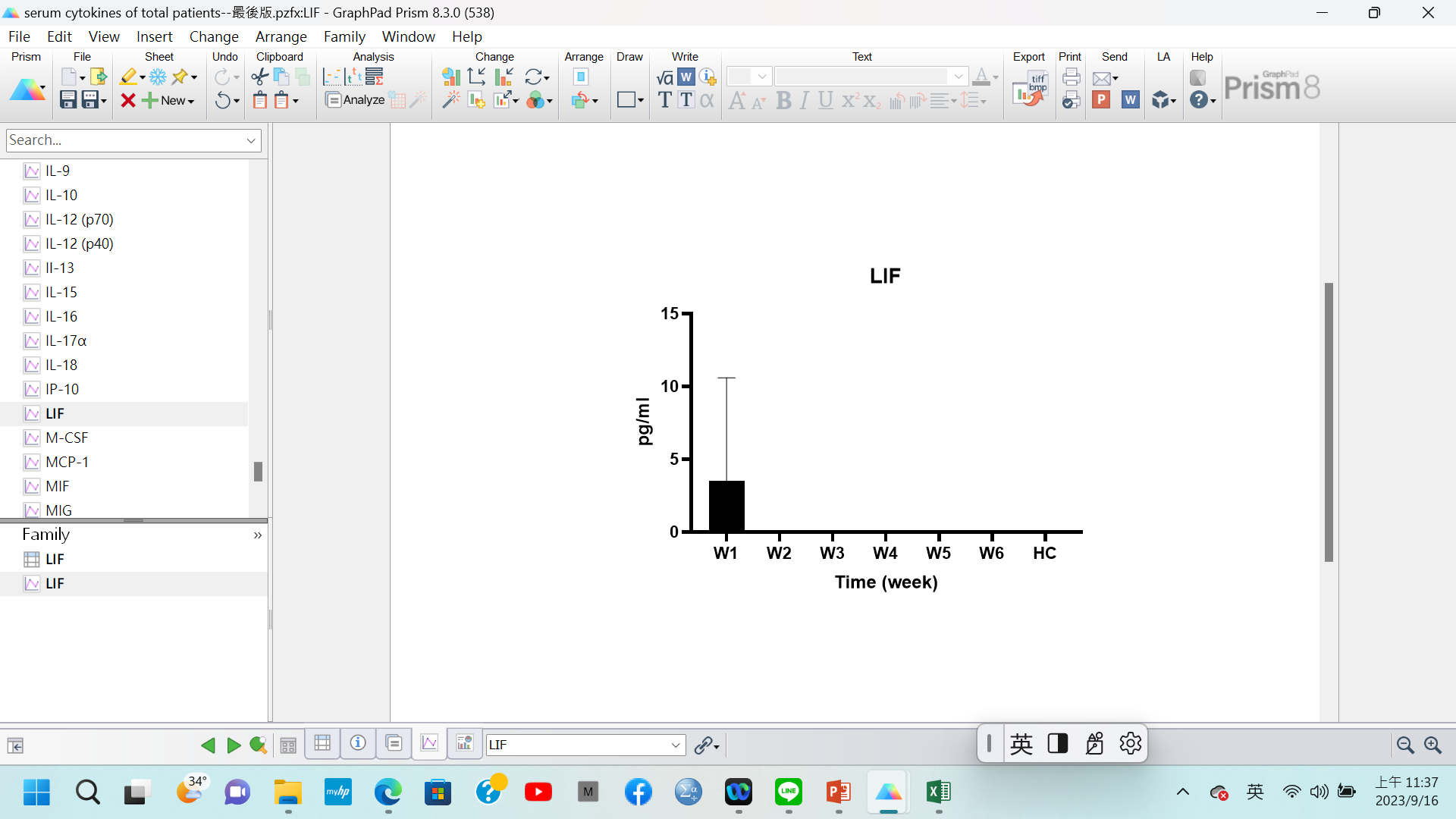


(P)


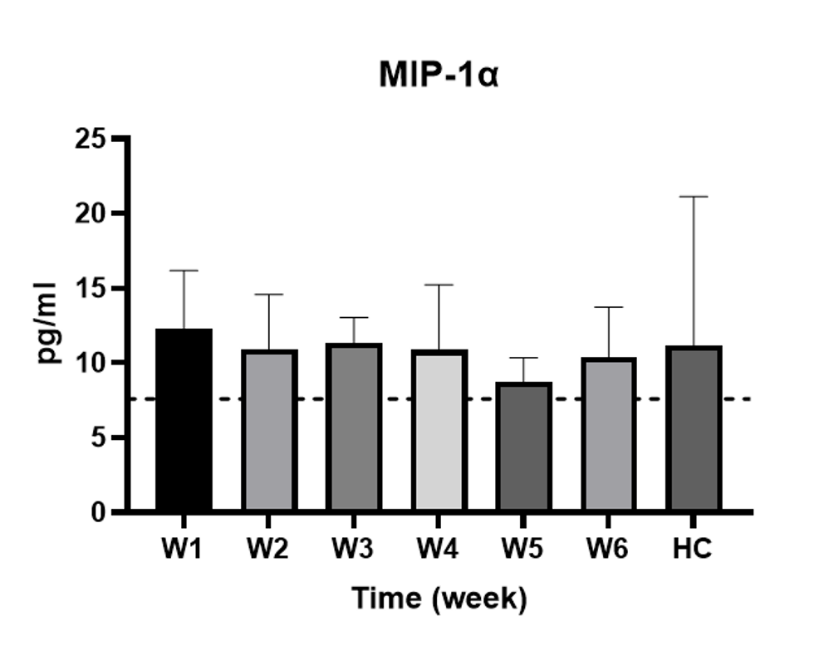


(Q)


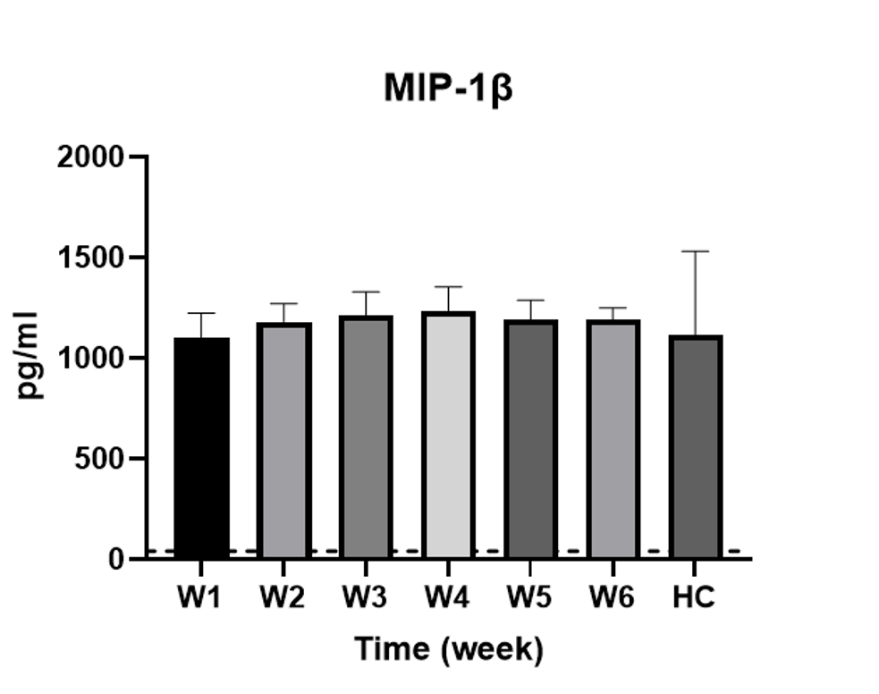


(R)

**Supplementary Figure 1.**

**Supplementary Figure 1.**

Serum cytokines without elevation in patients with COVID-19 over weeks 1–6 after symptom onset. The levels of each cytokine in healthy subjects are indicated as dotted lines.
